# Loss of function mutations in essential genes cause embryonic lethality in pigs

**DOI:** 10.1371/journal.pgen.1008055

**Published:** 2019-03-15

**Authors:** Martijn F. L. Derks, Arne B. Gjuvsland, Mirte Bosse, Marcos S. Lopes, Maren van Son, Barbara Harlizius, Beatrice F. Tan, Hanne Hamland, Eli Grindflek, Martien A. M. Groenen, Hendrik-Jan Megens

**Affiliations:** 1 Animal Breeding and Genomics, Wageningen University & Research, Wageningen, the Netherlands; 2 Norsvin SA, Hamar, Norway; 3 Topigs Norsvin Research Center, Beuningen, the Netherlands; 4 Topigs Norsvin, Curitiba, Brazil; University of Bern, SWITZERLAND

## Abstract

Lethal recessive alleles cause pre- or postnatal death in homozygous affected individuals, reducing fertility. Especially in small size domestic and wild populations, those alleles might be exposed by inbreeding, caused by matings between related parents that inherited the same recessive lethal allele from a common ancestor. In this study we report five relatively common (up to 13.4% carrier frequency) recessive lethal haplotypes in two commercial pig populations. The lethal haplotypes have a large effect on carrier-by-carrier matings, decreasing litter sizes by 15.1 to 21.6%. The causal mutations are of different type including two splice-site variants (affecting *POLR1B* and *TADA2A* genes), one frameshift (*URB1*), and one missense (*PNKP*) variant, resulting in a complete loss-of-function of these essential genes. The recessive lethal alleles affect up to 2.9% of the litters within a single population and are responsible for the death of 0.52% of the total population of embryos. Moreover, we provide compelling evidence that the identified embryonic lethal alleles contribute to the observed heterosis effect for fertility (i.e. larger litters in crossbred offspring). Together, this work marks specific recessive lethal variation describing its functional consequences at the molecular, phenotypic, and population level, providing a unique model to better understand fertility and heterosis in livestock.

## Author summary

Lethal recessives are mutations that cause early lethality in homozygous state that usually occur at very low frequency in wild and domestic populations. In livestock, however, those mutations might become more prevalent as a result of inbreeding. In this study, we report five such recessive lethal haplotypes that cause embryonic lethality in homozygous state in pigs. The causal mutations are of different type but all destroy the structure of essential genes involved in cellular housekeeping processes, essential for embryonic development. The lethal recessives have substantial impact on the population fitness affecting up to 3% of the population litters, causing the death of 0.52% of the total population of embryos. Moreover, these 'natural knockouts' can increase understanding of gene function within the mammalian clade. Together, our study will allow monitoring, and facilitate the purging and partial elimination of recessive lethal mutations in frequently used pig breeds.

## Introduction

Lethal recessive alleles cause pre- or postnatal death in homozygous affected individuals, reducing fertility in various populations [1]. Although recessive lethals are generally widespread throughout populations, their effect is generally masked by the extremely low frequency of individual mutations. However, within small sized domestic and wild populations, those alleles might be exposed by inbreeding [2, 3], caused by matings between related parents that inherited the same recessive lethal allele from a common ancestor.

The precise impact of recessive lethals depends on the population structure (i.e. effective population size) and recessive lethal mutation rates. In livestock, populations have been subject to intensive (genomic) selection resulting in relative small effective population sizes [4]. With small effective population size, genetic drift can rapidly increase the frequency of recessive lethals in the population. Although genomic selection has enabled substantial improvement on various traits including production, fertility, and disease resistance [5], it does not provide much advantage over traditional selection when it comes to controlling the frequency of recessive lethal mutations [6].

Several studies have reported recessive lethal variation (i.e. death of embryo or foetus prior to birth), likely derived from a single sire origin, to be maintained in livestock populations [1, 7]. In fact, the frequency of some recessive lethals were driven by heterozygote advantage for important production traits, e.g. milk yield in cattle [8], or growth in pigs [9], although the majority was likely the result of genetic drift. Together these studies show that lethal recessive alleles can have a considerable impact on population fitness, emphasizing the need for early detection. Although various recessive embryonic lethal loci have been reported in livestock, pinpointing the causal mutation can be extremely difficult. Charlier et al (2016) showed, using a reverse genetic screen, that loss-of-function mutations and deleterious missense mutations cause embryonic lethality in cattle populations. Nevertheless, the discovery of recessive embryonic lethals is often hampered by the lack of affected individuals and the relative low frequency. Genotyping and sequencing large cohorts of animals within single populations can therefore facilitate the discovery of such detrimental variation, and point directly to the causal mutations.

Pig fertility has increased steadily over the past years [10]. Breeding for improved fertility concerns a large number of traits with a combined effect on overall fertility, and lethal recessives are increasingly considered to substantially affect fertility in purebred livestock populations [11]. However, in pigs, the final production animals are crossbreds between purebred populations, usually derived from three-way crosses [12, 13]. First, crossbred sows are created from two elite purebred populations selected for high production of piglets (i.e. ‘maternal lines’), which then are crossed with a third elite purebred population especially selected for meat production traits (i.e. ‘paternal line’). These crossbreds are known to perform better on multiple traits compared to their parental purebred lines, in particular for traits related to fertility and robustness [14], as a result of the heterosis effect. Heterosis is caused by different non-additive effects, such as dominance, and it has been subject to a scientific controversy; the dominance hypothesis emphasizes the suppression of undesirable recessive alleles (by dominant alleles), while the overdominance hypothesis emphasizes on heterozygote advantage [15]. However, the magnitude of recessive lethals contributing to heterosis is largely unknown.

In this study we aim to explore the impact of lethal recessive variation in two pig populations using the following stepwise approach (1) perform simulations to assess the impact of genetic drift on lethal recessives, (2) identify haplotypes harboring lethal alleles using large-scale genotype data as developed by VanRaden et al. [16], (3) confirm lethality by reduced fertility in carrier animals, (4) identify causal mutations segregating on these haplotypes using whole genome sequence data (WGS) and RNA-sequencing data, (5) study the impact of recessive lethals on heterosis for fertility related traits.

## Results

### Population genetics of recessive lethal alleles

#### Estimating the number of recessive lethals segregating in two pig populations

In this study we analysed large-scale genomics, transcriptomics, and phenotype data from two commercial pig populations (Landrace and Duroc) to study recessive lethal alleles. We first evaluated the expected number and average frequency of recessive lethals within these two populations. Both the number and average frequency is a function of recessive lethal mutation rates and effective population size. The pig populations under study have an effective population size (Ne) in the range 100–150 [17]. Assuming similar mutation rates (~0.015 recessive lethals per gamete) as described for humans [18] and cattle [19], we estimate that about 20 recessive lethal alleles are segregating (at average 2% allele frequency) in each of the pig populations under study. This corresponds to about one recessive lethal allele carried per individual and the death of 1% of the embryos in the population as a result of homozygosity for a recessive lethal allele [19].

#### Simulating the impact of genetic drift on recessive lethals

The impact of genetic drift on recessive lethals heavily depends on the population structure and Ne. Small Ne leads to high extinction rates of *de novo* recessive lethals, but the few that are not lost tend to spread and increase in frequency. However, at a certain frequency, a trade-off between drift and selection is reached, at which the loss of homozygotes will prevent further increase assuming no heterozygote advantage. We evaluated this trade-off value (i.e. the maximum allele frequency reached by drift) using the actual population structure of the pig populations under study. We simulated the allele frequency change of a recessive lethal allele (fitness of homozygote mutants set to 0) over 25 generations in 1000 replicate populations with different start frequencies (**S1 and S2 Figs,** see Methods for details). Across simulations, the median frequency declines slightly with time (**S1 Fig**), but the decline is slower at lower allele frequencies (**S2 Fig**), due to very low number of carrier-by-carrier matings exposing the negative fitness effect. Interestingly, at about 10% allele frequency, the loss of homozygotes seems to prevent further increase of the allele frequency in the population (**S1 and S2 Figs**). This upper boundary is not observed under neutral assumptions (no negative fitness effect), in which the allele frequency can rise up to 30–40% within 25 generations (**S3 Fig**). Together, these results show that lethal alleles can reach allele frequencies up to 10% (20% carrier frequency) by genetic drift alone, although this happens only for a small fraction of the lethal variants.

In addition, we studied what proportion of the *de novo* recessive lethal mutations, is expected to remain in the population after 10 generations despite their very low starting frequency (0.024%). From the total number of *de novo* mutations, we show that about 2% still segregates after 10 generations (**S4 Fig**), and 1% after 25 generations (**S5 Fig**). We observe a similar pattern for neutral and recessive lethal *de novo* mutations, as there is very little purging efficiency at very low allele frequency (<2%).

### Detection of haplotypes harbouring lethal recessives segregating at moderate frequencies in two purebred pig populations

**Fig 1:**
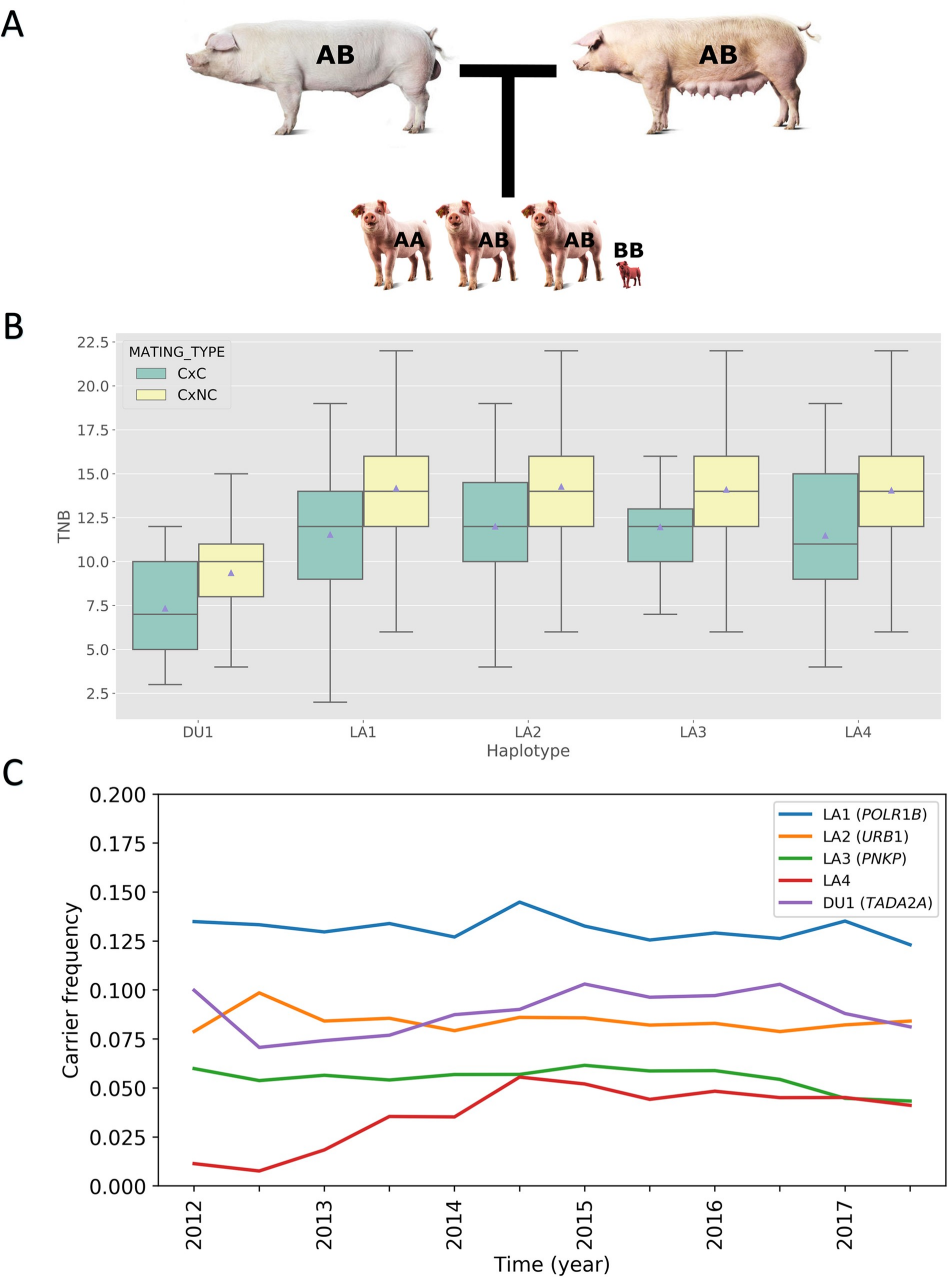
**A) Example of a carrier-by-carrier mating in Landrace.** CxC litters will result in a 1:2 genotype ratio instead of the normal 1:2:1 genotype ratio. **B) Fertility phenotypes for lethal recessives.** A significant reduction in total number born (TNB) is observed for CxC compared to CxNC matings. **C) Carrier frequency for lethal alleles in the period 2012–2018.** Figure shows relative stable carrier frequencies over a time period of 6 years (except for LA4).

To identify lethal alleles segregating in the pig populations we examined genotype data from 28,085 (Landrace), and 11,255 (Duroc) animals. All animals were genotyped or imputed to a medium-density 50K SNPchip (**S1 Table**). The genotypes were phased to build haplotypes, and then we applied an overlapping sliding window approach to identify haplotypes that show a deficit in homozygosity, likely harbouring a lethal recessive allele [16]. The analysis yielded one strong candidate haplotype (DU1) harbouring a lethal recessive allele in the Duroc population, and four candidates in the Landrace population (LA1-4), respectively (**Table 1**). Haplotype lengths range from 0.5 to 5 Mb and carrier frequencies range from 4.6 to 13.4%. We observe no homozygotes for DU1, LA1, and LA3 haplotypes, while we expected 26, 126, and 16, respectively. We do observe two, and three homozygotes for LA2 (50 expected) and LA4 (14 expected), suggesting incomplete linkage disequilibrium (LD) of the haplotypes with the causal lethal recessive mutation. Four out of five haplotypes show deviation from Hardy-Weinberg equilibrium with over 50% carrier offspring for carrier-by-carrier matings. This is in concordance with the absence of homozygous offspring, resulting in a 1:2 offspring ratio instead of the expected 1:2:1 genotype offspring ratio (**Fig 1A**, **Table 1**).

**Table 1:** Haplotypes exhibiting missing or deficit homozygosity. Table shows five loci exhibiting missing or deficit homozygosity on the Sscrofa11.1 genome build, four in the Landrace population (LA1-4), and one in the Duroc population (DU1). The table shows the genomic location, carrier frequency, and deficit of homozygosity for each haplotype. The deficit of homozygosity is calculated based on trio information (parents and offspring) with the formula described by Fritz et al., 2013 [20], and from haplotype frequency, using the Hardy-Weinberg principle. Genotyped progeny is derived from CxC matings.

Hap.	SSC	Start	End	#Carriers	Carrier. Freq	Expected (trio)	Expected (freq.)	Observed	Exact binomial test	# Genotyped progeny	# Heterozygote progeny
**DU1**	12	38.5	39.0	1,084	9.6	7 .0	26.1	0	1.81e-13	28	18 (64.3%)
**LA1**	3	42.6	47.5	3,763	13.4	52.0	126.0	0	2.11e-63	208	120 (57.7%)
**LA2**	13	195.7	196.2	2,358	8.4	18.25	49.5	2	6.39e-22	73	53 (72.6%)
**LA3**	6	52.5	54.0	1,319	4.7	6.0	15.5	0	2.54e-08	24	11 (45.8%)
**LA4**	12	25.0	27.0	1,271	4.6	-	14.4	3	0.00017	5	3 (60%)

### Carrier-by-carrier matings produce significantly smaller litters

We analysed the effect of the haplotypes on fertility phenotypes including total number born (TNB), number born alive (NBA), number of stillborn (NSB), and number of mummified piglets (MUM). We examined a total of 504 carrier-by-carrier (CxC) and 5,992 carrier-by-noncarrier (CxNC) matings (**Table 2**). Interestingly, all five haplotypes show significant reduction in both TNB (**Table 2, Fig 1B**) and NBA for CxC matings (**S2 Table**). The reduction in TNB ranges from 15.1 to 21.6% which is somewhat smaller than the expected 25% assuming early lethality with complete penetrance for homozygotes (**Table 2**). No significant increase in number of stillborn (NSB) or mummified piglets (MUM) was found, suggesting that homozygotes die very early in pregnancy (**S3 Table**). Together the 504 CxC matings cause a loss of 1,261 piglets over the last 5 years (comparing average litter size of CxC and CxNC matings), affecting 2.9% and 0.92% of all litters in the Landrace and Duroc population, respectively (**Table 2**). None of the five regions were previously reported to be associated with reduced TNB [21].

**Table 2:** Fertility phenotypes for total number born. Table shows the number of CxC and CxNC mating for each haplotype, the reduction in total number born (TNB), the percentage of affected litters in the population, the piglet loss associated with the CxC matings, the percentage of embryo deaths in the entire population, and the overall population piglet reduction.

Population	Hap.	#CxC	#CxNC	TNB (CxC)	TNB (CxNC)	Reduction	% Affected litters	Piglet loss	% Death	Population piglet reduction*
Landrace	LA1	297	2,350	11.51	14.18	18.8%	1.796	792.99	0.338	0.0479
	LA2	127	1,527	12.00	14.26	15.9%	0.706	287.02	0.112	0.0159
	LA3	30	872	11.96	14.09	15.1%	0.212	63.90	0.032	0.0045
	LA4	29	950	11.48	14.05	18.3%	0.212	74.53	0.039	0.0055
	**SUM**	**483**	**5,699**	-	-	-	**2.926**	**1218.44**	**0.521**	**0.0739**
Duroc	DU1	21	293	7.33	9.35	21.6%	0.922	42.42	0.199	0.0186

* Calculated as the product of the average TNB (Landrace: 14.18, Duroc: 9.35) and the population deaths in the Landrace and Duroc population.

### Candidate embryonic lethal alleles predicted from whole-genome sequence (WGS) and RNA-sequencing (RNA-seq) data

To find causal mutations, we analysed WGS (Landrace: 167, Duroc: 119) and RNA-seq (Landrace: 34, Duroc: 25) data available from the populations under study (**S4–S5 Tables**). The data was mapped to the latest Sscrofa11.1 reference build and functionally annotated using the Variant Effect Predictor (VEP) [22]. Next, we focussed on variants likely causing embryonic lethality (EL) in homozygous state, examining the impact of individual variants on the proteins. First, we selected loss-of-function (LoF) variants (frameshift, stop-gained, splice-site) and predicted deleterious missense variants within each population [23]. The predicted LoF and deleterious mutations show clear patterns of purifying selection, as observed from generally lower allele frequencies (**S6 Fig**), an enrichment of inframe indels (**S7 Fig**), and an enrichment of LoF mutations in the N-and C-terminal end of the gene (**S8 Fig**).

### Identifying candidate LoF mutations in lethal haplotypes

The likelihood of carriers being present in even a small random sample of pigs is high due to the relatively high carrier frequency of the candidate haplotypes found in this study. The candidate haplotypes could therefore be identified in pigs of the same populations for which WGS data (LA1: 21, LA2: 17, LA3: 7, LA4: 9, DU1: 9) or RNA-seq data (LA1: 4, LA2: 3, DU1: 3) was available. For each of the five haplotypes we used criteria of physical distance and co-segregation (see Methods for details) to select candidate causal mutations. A single strong candidate mutation was identified for all haplotypes, except LA4 (**Table 3**).

**Table 3:** Candidate causal variants for lethal haplotypes. The table shows the type, location, the affected gene, and the predicted impact for each candidate recessive lethal variant. The relative position in the protein shows the position of the variant relative to the protein length, for splice-variants, the affected intron is presented.

Hap.	Type	SSC	Position	Ref	Alt	Gene	AA change	Relative pos. in protein	Gene name
DU1	Splice-donor	12	38,922,102	G	A	*TADA2A*	p.Ile319fs	Intron 13	Transcriptional adaptor 2A
LA1	Splice-region	3	43,952,776	T	G	*POLR1B*	p.Ile701fs	Intron 14	RNA polymerase I subunit B
LA2	Frameshift	13	195,977,038	C	-	*URB1*	p.Val1961fs	0.87	Ribosome biogenesis homolog
LA3	Missense	6	54,880,241	T	C	*PNKP*	p.Gln96Arg	0.17	Polynucleotide kinase 3'-phosphatase

#### A splice donor mutation in TADA2A induces embryonic lethality in Duroc (DU1 haplotype)

Whole genome sequence data from nine DU1 carrier animals revealed 20 variants in high LD (r^2^ > 0.8) with the DU1 haplotype (**S6 Table**), of which only one variant is predicted to have high impact. This variant, a heterozygous splice-donor mutation (12:g.38922102G>A) in the Transcriptional adapter-Ada2 (*TADA2A*) gene is in complete LD with the DU1 haplotype (**Table 3**). The mutation affects a conserved GT splice dinucleotide site at the 5’ end of the intron between exons 13 and 14 (**S9 and S10 Figs**). We evaluated the effect on RNA splicing using RNA-seq data from three carrier animals (**S5 Table**). The splice-donor mutation seems to cause retention of intron 13 between exon 13–14 in one of the samples (**S11 Fig**), shown by reads spanning the exon-intron boundaries on the splice donor and acceptor sites in intron 13, not seen for non-carriers. Interestingly, two other carrier samples show exon skipping of exon 13 (**S9 Fig**), resulting in a frameshift, the addition of a novel methionine, and a premature stop codon in the first codon of exon 14. The mutant mRNA codes for a truncated TADA2A protein (318 amino acids) lacking the terminal 101 amino acids (AA) that includes the conserved SWIRM domain required for DNA binding [24]. These results show that the splice-donor mutation affects *TADA2A* splicing with different consequences (both exon skipping and intron retention) in carrier animals, but in all cases result in a compromised, non-functional transcript. The TADA2A protein is involved in the general transcription machinery and it’s gene is known to be essential in yeast and drosophila [25]. However, no information for mice null-mutants is available for *TADA2A* [26].

#### A splice region mutation in POLR1B induces early embryonic lethality in Landrace (LA1 haplotype)

**Fig 2:**
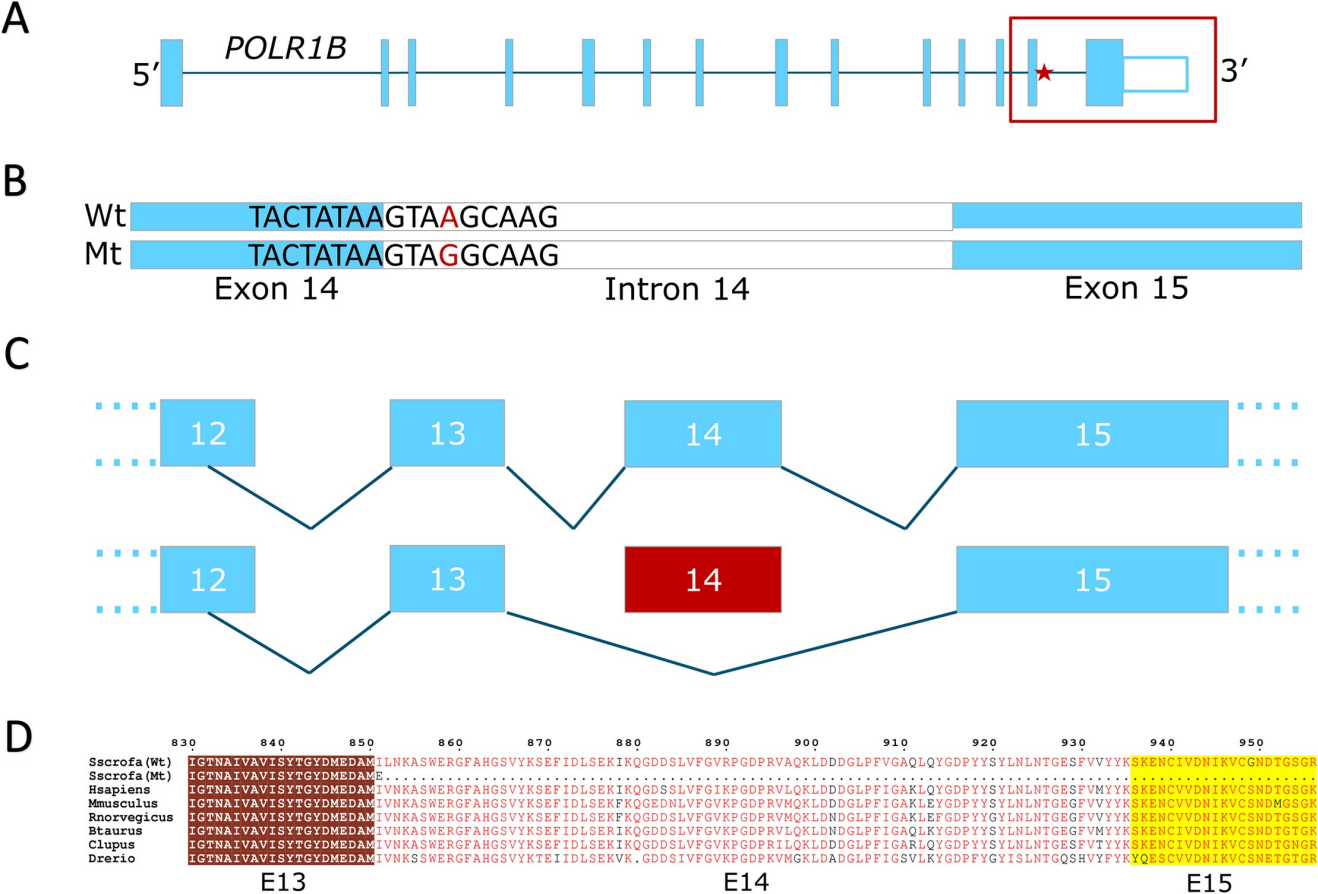
**A) *POLR1B* gene model.** The location of the mutation on the splice-donor site of intron 14 is indicated with a red star. **B) Illustration of the affected exon-intron splice region.** The causal 3:g.43952776T>G mutation is indicated in red. **C) Exon skipping of *POLR1B*.** The mutation causes complete exon skipping of exon 14, resulting in a truncated mRNA. **D) Alignment of the mutant (Mt) and wildtype (Wt) POLR1B protein sequence.** Skipping of exon 14 introduces a glutamic acid and a premature stop codon in the second codon of the terminal exon.

The LA1 haplotype was the longest haplotype observed in this study (SSC3:42.6–47.5). Therefore, we first performed a fine-mapping analysis to further pinpoint the region containing the causal mutation. We observed two recombinant animals (**S7–S8 Tables**) that were homozygous for a part of the LA1 haplotype in the region (45.6–47.5), leaving a final candidate region of length 3Mb (SSC3: 42.6–45.6 Mb). Whole genome sequence data from twenty-one LA1 carrier animals revealed a set of 415 variants, and one small intronic deletion in high LD (r^2^ > 0.8) with the LA1 haplotype (**S9 Table**), of which five variants are located within coding sequence (2 missense, 1 synonymous) or splice regions (2 splice-region). Both missense variants are predicted to be tolerated by SIFT, unlikely to be causal (**S9 Table**). However, the splice region mutation in intron 14 of the RNA polymerase I subunit B (*POLR1B*) gene is predicted to have high impact (**Table 3, S12 Fig**). The splice mutation affects a conserved adenine in the GTRAG splice site motif (positive strand: 3:g.43952776T>G, **Fig 2A and 2B**). The adenine is conserved throughout a wide range of vertebrate species (**S12 Fig**). Next, we analysed the RNA-seq data from four carrier animals and found that the splice region mutation causes exon skipping of exon 14 in all four carrier animals (**Fig 2C, S13 and S14 Figs**), not observed for non-carrier animals (**S15 Fig**). *POLR1B* isoforms that show alternative splicing for exon 14 have not been annotated in pigs or any other mammals, including human and bovine embryonic tissues. Skipping of exon 14 introduces a glutamic acid and a premature stop codon in the second codon of the terminal exon, lacking the final 370 amino acids located in the conserved subunit 2, hybrid-binding domain (binding to the DNA strand) (**Fig 2D**). Hence, this splice-region mutation likely causes a complete LoF of the POLR1B protein. The structure of RNA-polymerase 1, and the affected POLR1B subunit is presented in **S16 Fig**. The *POLR1B* gene is strongly conserved among vertebrates and null-mutant mice show embryonic lethality even prior to implantation [27].

#### A frameshift mutation in URB1 causes embryonic lethality in Landrace (LA2 haplotype)

Whole genome sequence data from seventeen LA2 carrier animals revealed a set of 234 variants and one small intronic deletion in high LD (r^2^ > 0.8) with the LA2 haplotype (**S10 Table**), of which five variants are located within coding sequence (1 frameshift, 1 missense, 2 synonymous) or splice regions (1 splice-region). The missense and splice-region variant in the ISTN1 gene are predicted to be tolerated, unlikely to be causal (**S10 Table**). However, the frameshift mutation in exon 38 of the *URB1* gene (13:g.195977038delC) caused by a 1-bp deletion is predicted to have high impact (**Table 3, S17 Fig**). The frameshift (ENSSSCP00000036505:p.Val1961fs) introduces 26 novel amino acids and a premature stop codon, producing a truncated protein of 1,986 amino acids, lacking the final 261 amino acids compared to the wild-type protein (2,247 AA). *URB1* (Ribosome Biogenesis 1 Homolog) is involved in the biogenesis of the 60S ribosomal subunit and is an essential gene in yeast and drosophila [28], but no information from mouse null-mutants is available for this gene. Moreover, no homozygous LoF mutations are reported in the ExAc database for the *URB1* gene [29], supporting that a functional copy is required.

#### A missense mutation in PNKP is a candidate to cause embryonic lethality in Landrace (LA3 haplotype)

Only four variants are found to be in high LD with the LA3 haplotype (**S11 Table**) including one deleterious missense mutation in the *PNKP* gene (6:g.54880241G>T), predicted to be strongly deleterious by SIFT (0.02) and PROVEAN (-2.9). The missense mutation causes a glutamine to arginine amino acid substitution (ENSSSCP00000003467:p.Gln96Arg) (**Table 3, S18 Fig**). The glutamine residue is highly conserved among vertebrates (**S19 Fig**), and is part of the protein-protein interaction FHA and SMAD domain. The *PNKP* gene plays a key role in the repair of DNA damage, being an essential part in the non-homologous end-joining (NHEJ) and base excision repair (BER) pathways. Mouse null-mutants exhibit embryonic lethality [30]. However, homozygous loss-of-function mutations in PNKP are associated with various neurologic diseases in human, but not with early lethality [31].

### Nonsense mediated decay of alternatively spliced transcripts

We assessed whether the splice mutations in *TADA2A* and *POLR1B* are subject to nonsense-mediated mRNA decay, a surveillance pathway eliminating transcripts that contain premature stop-codons [32]. We assessed the expression of the wild-type and mutant transcripts in carrier animals for both genes. The abundance of both the mutant *TADA2A*, and *POLR1B* transcripts are significantly lower (2.5- to 5 fold) compared to the wild-type transcripts, supporting that the mutant transcripts are likely subject to nonsense mediated decay (**S12 Table**).

### Validation of candidate causal mutations in carrier-by-carrier litters

We genotyped the complete litters of three LA1, and one LA2 CxC mating for the predicted causal mutations, and confirmed the carrier status of both parents for each litter (**S13–S14 Tables**). The three LA1 litters produced 38 piglets, 14 were homozygous for the wild-type allele (36.8%), 24 were heterozygous carriers (63.2%), and no homozygous mutants were found (P<0.005, **Table 4**). The LA2 litter produced 13 piglets, 3 homozygous wild-type, 10 heterozygous carriers of the deletion, and no homozygous del/del mutants (P = 0.076). These results are in line with the 1:2 genotype ratio expected for CxC litters, supporting the recessive lethality of the candidate causal mutations.

**Table 4:** Genotyping of causal mutations in four carrier by carrier litters. The parents (sow and boar) and complete liveborn and stillborn progeny are genotyped for the candidate causal mutations. Table shows the number of progeny, type of birth, and genotypes for the four examined litters.

LitterID	Haplotype—Gene	Gene—Mutation	# Progeny	# Liveborn	# Stillborn	# Wt	# Carrier	# Lethal	*p* (Chi-Square)
1	LA1-*POLR1B*	*POLR1B* 3:g.43952776T>G	14	13	1	TT = 4	TG = 10	GG = 0	
2	LA1-*POLR1B*	*POLR1B* 3:g.43952776T>G	11	11	0	TT = 3	TG = 8	GG = 0	
3	LA1-*POLR1B*	*POLR1B* 3:g.43952776T>G	13	12	1	TT = 7	TG = 6	GG = 0	
		**SUM—LA1**	**38**	**36**	**2**	**TT = 14**	**TG = 24**	**GG = 0**	**p<0.005**
4	LA2-*URB1*	*URB1* 13:g.195977038delC	13	11	2	CC = 3	C/Del = 10	Del/Del = 0	p = 0.076

### Embryonic lethal alleles are generally maintained at stable population frequencies

The current frequency of the embryonic lethals raises the question how population frequencies of lethal alleles have developed over the past years. Interestingly, we observe that overall the recessive lethal alleles are maintained at relative stable frequency over the past seven years (2012–2018, **Fig 1C**), despite ongoing selection on littersize in these populations.

#### Large-scale phenotype data supports both balancing selection and genetic drift driving the frequency of recessive lethals

High frequency of a lethal allele can be caused by a trade-off between a negative trait (i.e. reduced fertility) and another trait, e.g. improved growth [9]. We tested whether carriers of lethal haplotypes show signs of heterozygote advantage on one of the traits included in the breeding goal, which could potentially drive the frequency of the allele. However, we only found strong association signals for the most frequent haplotype LA1 (**S15 Table**). The carriers of the haplotype LA1, compared to non-carriers, show: increased mothering ability (fewer piglet deaths, and larger piglet weight at 21 days), increased carcass quality (larger loin depth, less backfat, and higher meat percentage), lower meat quality (less intramuscular fat), and slower growth (**S15 Table**). As expected, only a small negative effect on total number born is observed, as reduced litters will only be expressed in CxC matings. Mothering ability (consisting of several maternal traits) is a very important part of the breeding goal for this breed, and could be a strong candidate to support heterozygote advantage for this group of traits. Sows that carry the LA1 haplotype show lower piglet mortality at 21 days, and increased piglet weight at 21 days. For the other haplotypes, we only obtain weak associations (**S16 Table**), suggesting that the current frequency of the haplotypes are likely the result of genetic drift rather than heterozygote advantage.

### Lethal recessives explain part of the heterosis effect on fertility traits in the crossbred litters

The impact of individual lethal recessive alleles largely depends on its frequency. For example, assuming random matings, we estimate that about 1.8% of the population litters are CxC matings for LA1, while only 0.21% of the litters are CxC matings for the LA4 allele (**Table 2**). The four Landrace lethals combined affect 2.9% of the litters within the population, responsible for the death of 0.52% of the total population of embryos, which causes an average reduction of 0.073 TNB in the population (**Table 2**). Next, we investigated whether ELs could contribute to the heterosis effect for fertility (TNB) observed in the crossbreds. The current Landrace population is mostly crossed with a Large White (LW) population to generate a commercial F1 population. F1 litters in Landrace sows produce on average 0.20 piglet larger litters compared to purebred Landrace (LR = 14.18, LR/LW = 14.38) (**S17 Table**). All three identified mutations (LA1-LA3) are not segregating in the LW population, suggesting no homozygous affected individuals in the F1 population. Therefore, part of the TNB difference is likely caused by the four recessive lethals affecting the purebred litters (given the average reduction of 0.073 TNB as a result of the four ELs). Nevertheless, other heterotic effects will contribute to the increased litter size as well.

## Discussion

In this study we report five embryonic lethal haplotypes that segregate with carrier frequencies in the range of 4.6–13.4% in two commercial pig populations. We show that the use of large-scale genotype data within single populations provides the power to find lethal alleles with low frequencies. The inclusion of over 28 thousand individuals from the Landrace population, for instance, allowed us to detect the LA4 haplotype that has an allele frequency of only 2.3%. For three of the five recessive lethal haplotypes no homozygous carrier individuals were found, suggesting complete LD with a causal, recessive lethal variant. However, none of the recessive embryonic lethal haplotypes resulted in the theoretically expected 25% reduction in piglets born (range observed is: 15.1–21.6%). The most likely explanation is that the number of embryos frequently exceeds the uterine capacity of the sow. Hence, by reducing the number of embryos by 25%, fewer wildtype/wildtype and wildtype/mutant embryos are eliminated [33]. This compensatory effect could be especially relevant if homozygous affected zygotes fail to develop or embryos die very early on. Especially if they die prior to implantation in the uterus, other viable healthy zygotes can compensate (i.e. take their place in the uterus) of the lethal effect in homozygous zygotes. A compensatory effect is particularly likely for LA1 homozygous affected embryos (*POLR1B*), since in homozygous *POLR1B* knock-out mice embryos terminate development before implantation in the uterus is established [27]. Moreover, we did not observe an increase in mummified or stillborn piglets for CxC matings, again suggesting early termination (i.e. prior to day 35 in gestation) of homozygous animals in utero.

All four genes affected by embryonic lethal alleles are involved in cellular housekeeping functions including transcription (*POLR1B*, *TADA2A*), translation (*URB1*), and DNA damage repair (*PNKP*), supported by the relative high expression of these genes within different tissue types [34]. The RNA-seq data from carrier animals confirmed the functional impact of the DU1 splice-donor and LA1 splice-site mutations, both resulting in truncated proteins caused by the skipping of complete exons. Interestingly, we show that a single splice-donor mutation can simultaneously cause exon skipping and intron retention, something described previously in human studies [35], but not previously observed in pigs. Moreover, the alternatively spliced mRNAs are likely subject to nonsense-mediated decay, because the level of the mutant mRNA is significantly lower compared to the wild-type mRNA. All mutations (except *URB1*) are located within parts of the genes predicted to be intolerant to LoF mutations observed from a negative subRVIS score [36]. Interestingly, embryonic lethality has been described in targeted mice null-mutants for *POLR1B* and *PNKP* [27, 30], but not for *URB1* and *TADA2A*. In this study, however, we demonstrate that both *URB1* and *TADA2A* are essential for normal embryonic development in pigs, likely to be similar in human. We did not find any coding variants or structural variants that are in high LD with the LA4 haplotype (**S18 Table**). However, other type of variants (e.g. small insertion elements) could also induce embryonic lethality or genetic disease [37], something not well explored in this study.

We show that the frequency of the lethal haplotypes over time, at least over the past seven years, is stable, suggesting that there is no strong selection against these recessive lethal variants. The population genetic analysis indicates that the observed frequencies of recessive lethal alleles found in this study, can be the result of genetic drift alone. Moreover, the study on *de novo* mutations shows that the lethal mutations in the LA1, LA2, and DU1 haplotypes (allele frequency > 4%) likely arose over 25 generations ago (assuming no heterozygote advantage). Genetic drift as a driving force for the observed frequencies is further supported by the lack of clear evidence for heterozygote advantage (except for LA1). Nevertheless, we cannot exclude that these alleles have been subject to genetic-hitchhiking in the past, resulting in heterozygote advantage due to LD with a beneficial allele that became fixed.

Evidence for heterozygote advantage has been found for other highly detrimental variants that occur in higher frequencies than those observed here in wild and domesticated populations [8, 9]. This could also be the case for the most frequent recessive lethal in our study, LA1, for which a highly positive effect on mothering ability for heterozygous carriers was found. In sow lines, mothering abilities are among the most important selection traits. The favorable phenotype of heterozygous carriers (mothering ability) offsets the occasional lower litter size, as long as the carrier frequency does not become too high. Nevertheless, our simulations show that the allele frequency of recessive lethals can rise up to 10% as a result of genetic drift alone. At this frequency, the negative effects on fitness (i.e. smaller litters and lack of homozygotes) will prevent further increase in allele frequency.

Recessive lethals, by definition, deviate from the Hardy-Weinberg equilibrium (HWE). We analyzed whether our liberal HWE marker threshold might hampered the detection of high frequency ELs, but no new high frequency haplotypes were revealed (**S21 Table**). Nevertheless, not all embryonic lethal variation currently present in the populations under study was identified. In fact, even if LD between recessive lethal causal variants and SNP-chip based haplotypes would be perfect, the minimum allele frequency that could be detected is around 2% for the Landrace population, and around 4% for the Duroc population. In addition, lethal recessives residing on more common haplotypes cannot be detected because the SNP density is likely too low to distinguish between the haplotype with and the haplotype without the lethal recessive. We estimated that ELs likely account for 1% of deaths in these pig populations, but the four identified Landrace lethals account for the loss of 0.52% of all newborn pigs per generation, showing that the remainder 0.48% is caused by yet to be identified EL mutations.

In pigs, the crossbred production animals show clear signs of heterosis, especially for fertility related traits [14]. We provide compelling evidence that embryonic lethals contribute to the heterosis effect seen in the Landrace crossbred litters. Assuming that recessive lethal variation is generally occurring in a single breeding line only, crossbred products will only be heterozygous for the lethal recessive mutations. We show that at least 2.9% of the litters within a single pure breeding line (Landrace) are offspring of matings between carriers of lethal recessives identified in this study, and that the four identified lethal variants are responsible for a significant part of the total heterosis effect (as measured in surviving piglets). The heterosis effect is caused by the suppression of recessive lethal alleles by dominant wildtype alleles in the crossbreds [15], providing evidence that the impact of lethal recessives on fertility and heterosis in these commercial pig populations is likely underestimated. Nevertheless, other detrimental, but not lethal alleles, uniquely segregating in purebred pig populations likely contribute to the heterosis effect even more, although this has never been properly quantified.

Our study shows high resolution and efficiency of combining large-scale genotype (SNP chip), phenotype, whole-genome sequence, and RNA-sequencing data to identify deleterious mutations that confer early embryonic lethality in pigs. We report five relatively common embryonic lethal alleles with carrier frequencies between 4.7–13.4%. Four of the variants destroy the structure of essential genes involved in cellular housekeeping processes including mRNA transcription, translation, and DNA repair. Simulation shows that observed allele frequencies can be mainly explained as consequence of drift only and there is no clear evidence for heterozygote advantage for favourable traits. The large amount of phenotype and genotype data collected in modern breeding programs in combination with increasing genomic data provides excellent possibilities to monitor old and new detrimental mutations segregating in purebred livestock populations. Although, we provide compelling evidence that the identified embryonic lethal alleles contribute to the observed heterosis effect for fertility, only a small proportion of the overall heterosis can be explained by the effect of the EL alleles detected. Other factors contributing to heterosis remain to be detected.

## Methods

### Ethics statement

Samples collected for DNA extraction were only used for routine diagnostic purpose of the breeding programs, and not specifically for the purpose of this project. Therefore, approval of an ethics committee was not mandatory. Sample collection and data recording were conducted strictly according to the Dutch law on animal protection and welfare (Gezondheids- en welzijnswet voor dieren).

### Animals, genotypes and pre-processing

The dataset consists of 28,085 and 11,255 animals from Norwegian Landrace and Duroc purebreds, respectively. The animals are genotyped on the (Illumina) Geneseek custom 50K SNP chip with 50,689 SNPs (50K) (Lincoln, NE, USA). The chromosomal positions were determined based on the Sscrofa11.1 reference assembly. SNPs located on autosomal chromosomes were kept for further analysis. Next, the SNPs were filtered using following requirements: Each marker had a MAF greater than 0.01, and a call rate greater than 0.85, and an animal call rate > 0.7. SNPs with a p-value below 1x10^-5^ for the Hardy-Weinberg equilibrium exact test were also discarded. All pre-processing steps were performed using Plink v1.90b3.30 [38]. After quality control, the final dataset contained 43,375 and 42,706 markers for Landrace and Duroc populations, respectively.

### Phasing and identification of missing homozygote haplotypes

We used BEAGLE version 4.1 genetic analysis software to phase both populations separately [39]. Haplotypes exhibiting missing or deficit homozygosity were identified using an overlapping sliding window approach from 0.5 to 5 MB. Within each window individual haplotypes (with a frequency > 0.5%) were evaluated for missing or deficit homozygosity. The expected number of homozygotes was estimated using two methods: (1) Estimation based on haplotype frequency, using the Hardy-Weinberg principle, (2) Estimation based on haplotype information from both parental haplotypes with the formula described by Fritz et al., 2013 [20]. An exact binomial test was applied to test the number of observed homozygotes with the number of expected homozygotes. Haplotypes were considered significant if P < 5×10^−3^.

### Fine mapping of the LA1 haplotype

We examined the wild-type haplotypes for each LA1 carrier animal to identify recombinant individuals. We used PyVCF [40] to gather both haplotypes for all carriers animals within the LA1 genomic region from the BEAGLE phased VCF file. Next, we divided the LA1 haplotype in 5 shorter sub-haplotypes (length = 1Mb). Next, we examined whether the sub-haplotypes were carried in homozygous state in the group of LA1 carrier animals. Homozygous sub-haplotypes were excluded to carry the causal mutation.

### Phenotypic effects associated with lethal haplotypes

We examined phenotypic records for TNB, NBA, NSB, and MUM to verify the lethality of the detected haplotypes. We listed all CxC and CxNC matings available and used a Welch t-test to assess if the phenotypes from the CxC matings significantly differ from CxNC matings. A *P*-value < 0.05 was considered significant. The order of CxNC matings does not reflect the sex of the parent animal and is both carrier boar and carrier sow combined

### Population sequencing and mapping

Sequence data was available for 167 (Landrace) and 119 (Duroc) animals from paired-end 100 bp reads sequenced on Illumina HiSeq [41]. The sequenced samples are frequently used boars born between 2003 and 2017, selected to capture as much of the genetic variation present in the Landrace and Duroc populations. The majority of the sequenced animals were also represented in the 50K genotype dataset (Landrace = 161, Duroc = 72). The coverage ranges from 6.65 to 21.46, with an average coverage of 12.70 (**S19 Table**). Sickle software was used to trim the sequences [42]. BWA-MEM (version 0.7.15, [43]) was used to map the WGS data to the Sscrofa11.1 reference genome. Samtools dedup was used to discard PCR duplicates [15]. GATK IndelRealigner was used to perform local realignments of reads around indels [44].

### Variant discovery

Freebayes variant calling software was used to call variants with following settings: min-base-quality 10—min-alternate-fraction 0.2—haplotype-length 0—ploidy 2—min-alternate-count 2 [45]. Post processing was performed using bcftools [46]. Variants with low phred quality score (<20), low call rate (<0.7) and variants within 3 bp of an indel are discarded. Next, genotype calls are filtered for sample depth (min: 4, max: AvgDepth *2.5) leaving a total of 18,118,052, and 15,857,077 post-filtering variants for Landrace and Duroc population, respectively. The average variant call rate is 95.4% (Landrace) and 96.4% (Duroc), and the average transition / transversion (TS/ TV) ratio is 2.42 and 2.27, respectively, in concordance with previous findings in pigs [47].

### Structural variation

The Smoove pipeline (https://github.com/brentp/smoove) was used to call SVs. Smoove uses various software to call and filter SVs taking the alignment BAM files, and the Sscrofa11.1 reference genome as input. First, Lumpy software is used to call SVs [48]. Next, Svtyper is used to genotype SVs [49]. To further filter SV calls, Mosdepth is used to remove high coverage regions, and Duphold to annotate depth changes within and on the breakpoints of SVs.

### Functional annotation of variants

We performed variant (SNPs, Indels, and SVs) annotation using Variant Effect Predictor (VEP, release 90) [22]. The variant effect prediction in protein altering variants was performed using SIFT [23]. The following variant classes were considered potentially causing LoF: splice acceptor, splice donor, inframe indels, frameshift, stop loss, stop gained, and start lost variants.

### Candidate embryonic lethal alleles

LoF and deleterious missense variants were selected within each population that met following criteria. The variant is found in a maximum of 1 homozygous individual, allowing one false genotype assignment. Next, the variant is annotated in a gene that is a 1-to-1 ortholog with cattle to minimize the effect of off-site mapping of sequence reads, which can be particularly problematic for large gene families. Finally, the list of EL candidates was manually validated for possible sequencing and alignment artefacts. Further functional support was obtained from the MGI database release 6.10 (i.e. phenotypes from null-mutant mice) to predict the relative impact on the phenotype [26]. To identify candidate causal mutations for the haplotypes exhibiting missing homozygosity we applied the following criteria: 1) The mutation is located within 5 Mb of the haplotype boundaries. 2) The mutation is carried in heterozygote state by the haplotype carriers and no homozygous individuals are observed. 3) The mutation is absent from non-haplotype-carrier animals. 4). The mutation is in high LD with the candidate lethal recessive haplotype (R^2^ > 0.7). LD analysis was performed using Plink v1.90b3.30 [38] with following settings:—chr-set 18,—r2, ld-window-r2 0.7.

### RNA sequencing and nonsense mediated decay

The impact of the splice mutations on the expression of the gene was assessed using RNA-seq data. The animals sequenced are frequently used artificial insemination boars selected based on extreme phenotypes all present in the genotyping data [50]. The phenotypes are based on high and low sperm DNA fragmentation index, a measure of well packed double-stranded DNA vs single-stranded denatured DNA, which is an important indicator of boar fertility. We mapped the RNA-seq data to the Sscrofa11.1 reference genome using STAR [51] and called transcripts and FPKM expression levels using Cufflinks [52]. To test for nonsense mediated decay, we examined the transcript expression level of both the mutant and wild-type transcript identified by Cufflinks. The predicted effect on the mRNA was further evaluated by manually inspecting alignments using the JBrowse visualization software [53]. Variants were called on the RNA using Freebayes v1.1.0 [45] to examine if the genes are subject to genomic imprinting, heterozygous coding variants are listed in **S20 Table**.

### Validation of candidate causal mutations in carrier-by-carrier litters

We tracked four recent CxC litters and sampled the complete litter including parent animals. The complete litter and parents were genotyped for the candidate causal variants using matrix-assisted laser desorption/ionization time-of-flight mass spectroscopy (MALDI-TOF MS) assays. The candidate mutations were fitted into the same assay and the assay was designed using MassARRAY Assay Design software (Agena Biosciences, Hamburg, Germany). The genotyping was done using the IPLEX protocol according to manufacturer’s instructions. The difference in the expected and observed Mendelian genotype ratios was tested using a Chi-Square test.

### Frequency and impact of embryonic lethal alleles

We analyzed the frequency of the haplotypes harboring embryonic lethals per half-year starting from 01-jan-2012 and assessed the frequency on the total population (live animals) on each time point. We then examined the proportion of carrier and non-carrier animals to obtain the carrier frequencies for each time point. The percentage of affected litters was estimated by taking the product of the carrier frequency, and we examined the piglet loss using the phenotypic records available within the breeding program in the last seven years (2012–2018). To test whether the EL alleles contribute to the heterosis effect for fertility in the crossbred litters in purebred Landrace sows, we made following assumptions: First, we expected no EL litters in the crossbreds (heterozygotes). Second, we assumed 2.9% EL litters in purebred Landrace from the four identified lethal alleles. Third, we calculated the percentage of population deaths for each of the recessive lethals individually by taking the product of affected litters and the litter reduction. Combined, the four lethals account for 0.52% of population deaths, and the overall piglet reduction was calculated as the product of the average TNB (14.17) and the population deaths caused by EL litters in the Landrace population.

### Genetic drift simulation

We simulated changes in allele frequency across multiple populations under the model of Wright [54]. Each simulation was performed with different start frequencies, corresponding to the frequencies of the identified haplotypes. We selected a population Ne of 150, and population size of 2050 (50 boars, and 2000 sows). Each genotype has an associated fitness value, and we set the fitness to zero for homozygous lethal allele carriers, and fitness 1 (no negative fitness effect) to heterozygotes and non-carriers. We assume constant population size through time, and matings are simulated randomly at each generation. Changes in allele frequencies are calculated using the R package driftR (https://github.com/cjbattey/driftR). The simulation calculates allele frequencies from a random draw of a binomial distribution with a probability of success equal to the post-selection expected frequency for each generation and each population. The results are plotted in R using the package ggplot2 [55].

### *De novo* mutations

The frequency of *de novo* mutations was estimated based on a population size of 2050, accounting for a *de novo* mutation allele frequency equal to = 1/4100 = 0.024%. We used a human and cattle based per generation *de novo* mutation rate equal to 1.2^e-08^ per nucleotide per generation [56, 57]. The product of the genome size (in nucleotides) and mutation rate is used to calculate the number of *de novo* mutations per individual (4915.82 Mb * 1.2e-08 = 59). Considering a replacement rate of approximately 50%, we estimate that 60,475 *de novo* mutations will arise each generation (1,025*59). We used the same model from Wright [54] to simulate changes in allele frequency across multiple populations for *de novo* mutations.

### Breeding values and association analysis

To test whether carriers of lethal haplotypes show signs of heterozygote advantage on important traits in the breeding goal, we performed association analyses between all lethal haplotypes found in this study and a total of 25 traits (**S15–S16 Tables**) included in the breeding goal of the evaluated populations. Estimated breeding values (EBV) were used as a response variable for each trait under study. The EBV of each animal was obtained from the routine genetic evaluation by Topigs Norsvin using an animal model. Association analyses were performed using the software ASREML [58] applying the following model:

$$EBV_{ij}=\mu +H_{i}+a_{j}+e_{ij}$$

where *EBV*_*ij*_ is the observed EBV for the animal *j*, *μ* is the overall EBV mean of the population, *H*_*i*_ is the number of copies (0/1) of the lethal haplotype *i*, *a*_*j*_ is the additive genetic effect estimated using a pedigree-based relationship matrix, and *e*_*ij*_ the residual error. A p-value below 1 × 10^−5^ was considered significant.

## Supporting information

S1 Fig Genetic drift simulation for a lethal recessive with an allele frequency of 6.7% (13.4% carrier frequency) over 25 generations. (PDF)

S2 Fig Genetic drift simulation for a lethal allele with 2.3% allele frequency (carrier frequency: 4.6%) over 25 generations. (PDF)

S3 Fig Genetic drift simulation for a neutral allele with 6.7% allele frequency (13.4% carrier frequency) over 25 generations. (PDF)

S4 Fig Genetic drift simulation for a de novo mutation with start frequency of 0.024% over 10 generations. (PDF)

S5 Fig Genetic drift simulation for a de novo mutation with start frequency of 0.024% over 25 generations. (PDF)

S6 Fig Allele frequency distribution for synonymous, tolerated, and deleterious missense variants. (PDF)

S7 Fig Enrichment of inframe indels in coding regions. A) Indel length distribution for non-coding regions. B) Indel length distributions for coding regions.
(PDF)

S8 Fig Relative position in the protein for frameshift, non-frameshift, and stop-gained variants in the pig populations. (PDF)

S9 Fig Screen capture shows exon skipping of *TADA2A* exon 13 in two carriers (768945, 780181) of the DU1 splice-donor mutation. (PDF)

S10 Fig UCSC screen capture showing sequence conservation for the GT splice dinucleotide site in the TADA2A gene. (PDF)

S11 Fig Screen capture of intron retention for one DU1 carrier sample (906564) at the 5’ (A), and 3’ end (B) of *TADA2A* intron 13 caused by the DU1 splice-donor mutation.
(PDF)

S12 Fig Screen capture showing the splice region mutation in intron 14 of the *POLR1B* gene in one of the LA1 carrier animals (L330). (PDF)

S13 Fig A) UCSC screen capture of *POLR1B* splice region. B) Exon skipping of *POLR1B* exon 14 in one LA1 carrier animal.
(PDF)

S14 Fig Screen capture of exon skipping (indicated by grey lines) in the *POLR1B* gene (exon 14) caused by the 3:g.43952776T>G splice region mutation. (PDF)

S15 Fig Screen capture of three non-carrier animals of the LA1 splice-region mutation. (PDF)

S16 Fig Protein structure of RNA-polymerase 1. (PDF)

S17 Fig Screen capture of two carrier animals (L078, L362) for the LA2 (p.Val1961fs) frameshift mutation. (PDF)

S18 Fig Screen capture of one carrier animals (L827) for the LA3 missense mutation. (PDF)

S19 Fig PNKP multiple sequence alignment. (PDF)

S1 Table Number of genotypes and sequences individuals. (PDF)

S2 Table Phenotype records for CxC and CxNC matings (TNB: Total number born, NBA: Number born alive, NSB: Number stillborn, MUM: Number mummified). (PDF)

S3 Table Fertility phenotypes for liveborn, stillborn, and mummified piglets for CxC litters compared to CxNC litters. (PDF)

S4 Table WGS carrier animals for candidate lethal haplotypes. (PDF)

S5 Table Per-haplotype RNA-seq carrier animals. (PDF)

S6 Table Genomic variation in high LD with the DU1 haplotype. (XLSX)

S7 Table Wildtype and LA1 and recombinant haplotype in the region SSC1: 42.5–47.5. (PDF)

S8 Table Two recombinant samples used for fine-mapping of the LA1 haplotype. (PDF)

S9 Table Genomic variation in high LD with the LA1 haplotype. (XLSX)

S10 Table Genomic variation in high LD with the LA2 haplotype. (XLSX)

S11 Table Genomic variation in high LD with the LA3 haplotype. (XLSX)

S12 Table RNA-seq expression in fragments per kilobase per million (FPKM) for wild-type and mutant transcript. (PDF)

S13 Table Validation of LA1 causal mutation in three carrier-by-carrier litters. (PDF)

S14 Table Validation of LA2 causal mutation in one carrier-by-carrier litters. (PDF)

S15 Table Association analysis for LA1 carriers. (PDF)

S16 Table Association analysis for LA2, LA3, and DU1 carriers. (PDF)

S17 Table Litter information for purebred and crossbred litters in the Landrace population. (PDF)

S18 Table Genomic variation in LD with the LA4 haplotype. (XLSX)

S19 Table WGS samples including breed and coverage information. (PDF)

S20 Table Heterozygous variants identified in the *POLR1B* and *TADA2A* RNA-seq expression data. (PDF)

S21 Table Haplotypes detected using a strict Hardy-Weinberg exact test threshold for individual marker filtering (P < 1x10-30). (XLSX)
