# Supplementary material for: Loss of function mutations in essential genes cause embryonic lethality in pigs
Source: PLoS Genet. 2019 Mar 15;15(3):e1008055. doi: 10.1371/journal.pgen.1008055 (PMC6436757; doi:10.1371/journal.pgen.1008055)
Supplement: S3 Fig — (PDF) [file pgen.1008055.s003.pdf]

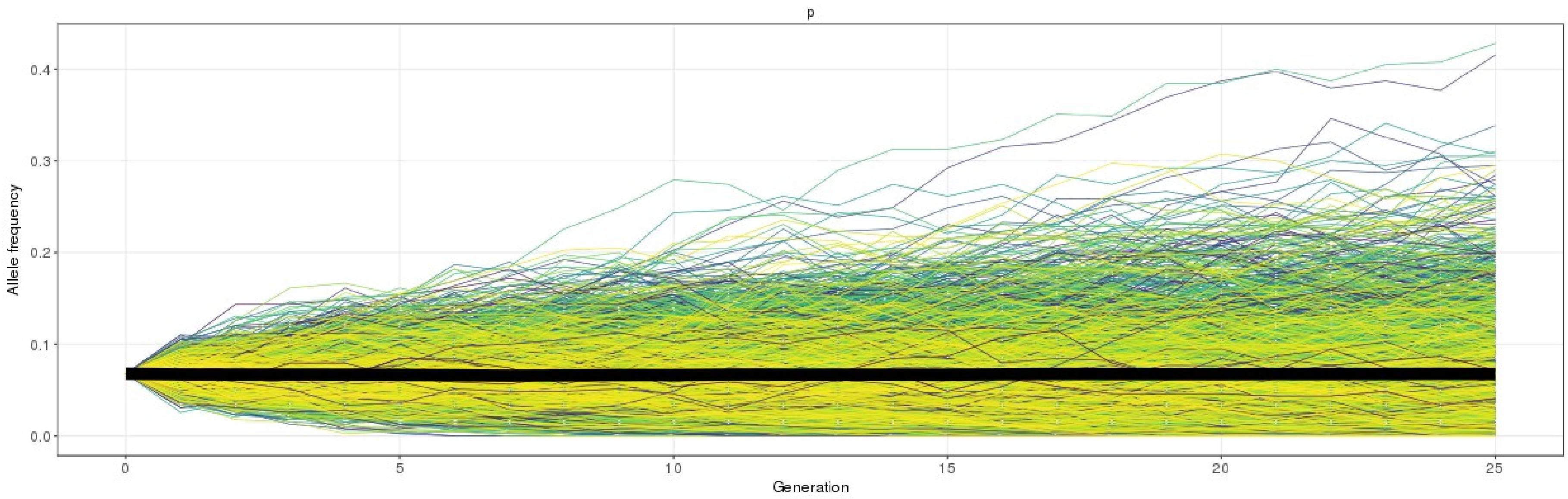

Figure S3: Genetic drift simulation for a neutral allele with 6.7% allele frequency (13.4% carrier frequency) over 25 generations. Plot shows frequency after 25 generations for 1000 simulations. The allele is lost in approximately 14% of the simulations, while in 49% of the simulations the frequency after 25 generations is equal or greater than the start frequency.
