# Supplementary material for: Loss of function mutations in essential genes cause embryonic lethality in pigs
Source: PLoS Genet. 2019 Mar 15;15(3):e1008055. doi: 10.1371/journal.pgen.1008055 (PMC6436757; doi:10.1371/journal.pgen.1008055)
Supplement: S4 Fig — (PDF) [file pgen.1008055.s004.pdf]

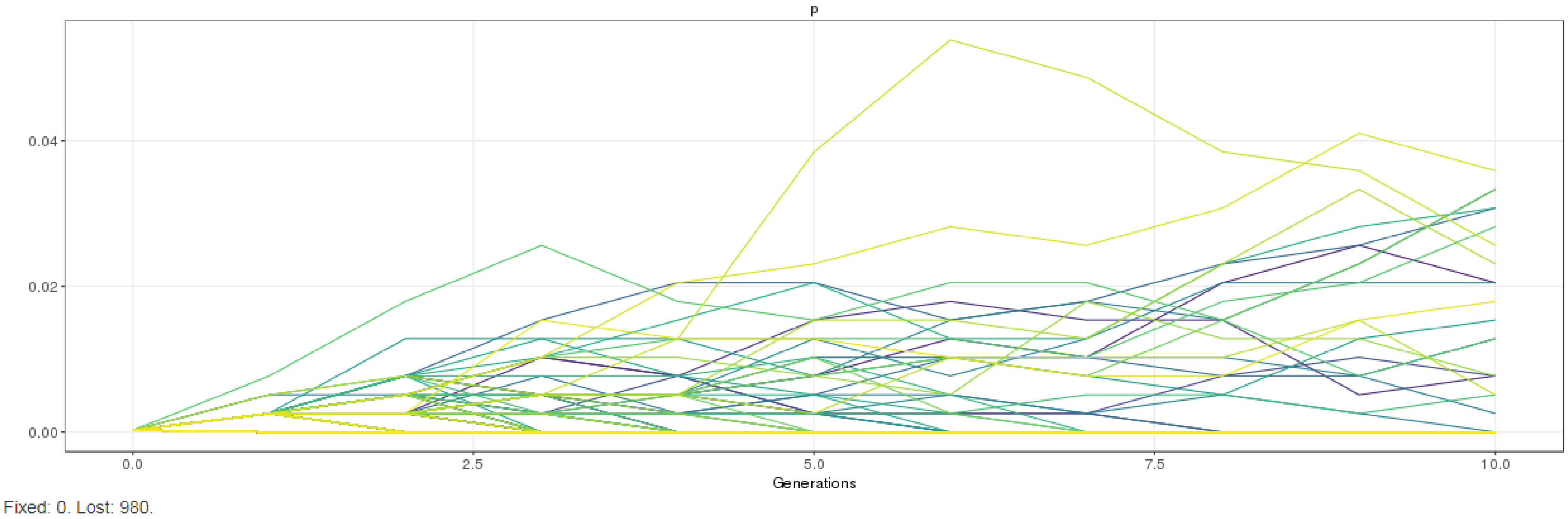


**Figure S4: Genetic drift simulation for a de novo mutation with start frequency of 0.024% over 10 generations.** Plot shows that in 20 out of 1000 simulations (2%) the de novo mutation is still segregating in the population after 10 generations.
