# Supplementary material for: Loss of function mutations in essential genes cause embryonic lethality in pigs
Source: PLoS Genet. 2019 Mar 15;15(3):e1008055. doi: 10.1371/journal.pgen.1008055 (PMC6436757; doi:10.1371/journal.pgen.1008055)
Supplement: S5 Fig — (PDF) [file pgen.1008055.s005.pdf]

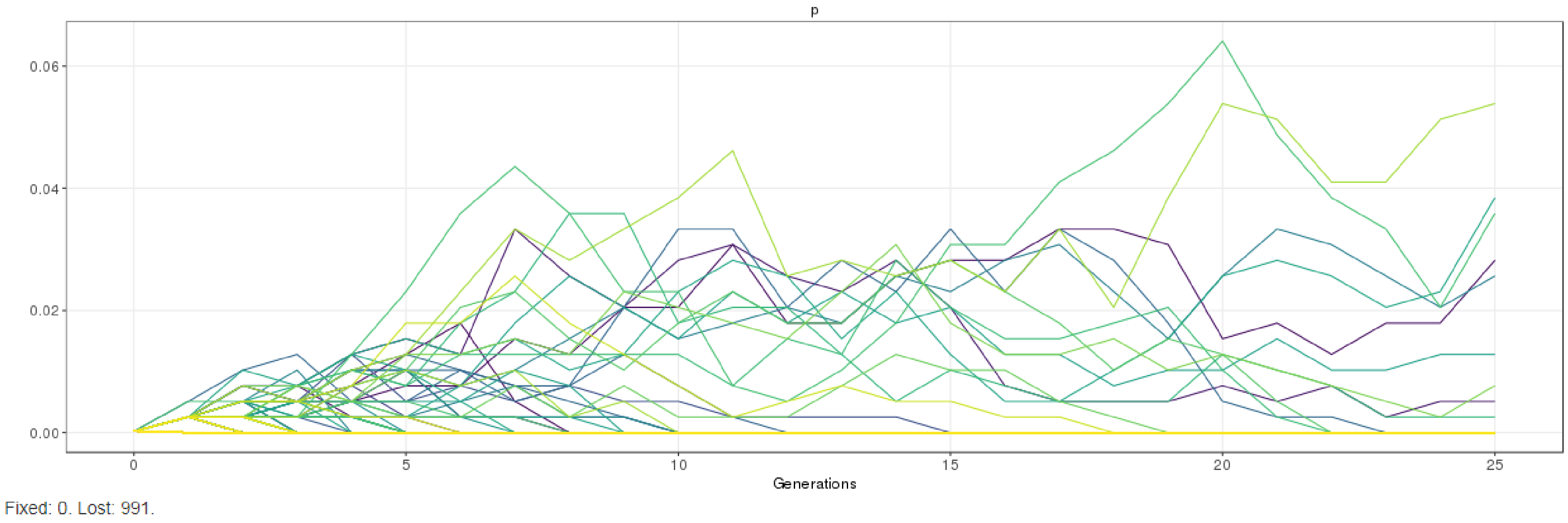

Figure S5: Genetic drift simulation for a de novo mutation with start frequency of 0.024% over 25 generations. Plot shows that in 9 out of 1000 simulations (0.9%) the de novo mutation is still segregating in the population after 25 generations.
