# Supplementary material for: Loss of function mutations in essential genes cause embryonic lethality in pigs
Source: PLoS Genet. 2019 Mar 15;15(3):e1008055. doi: 10.1371/journal.pgen.1008055 (PMC6436757; doi:10.1371/journal.pgen.1008055)
Supplement: S6 Fig — (PDF) [file pgen.1008055.s006.pdf]

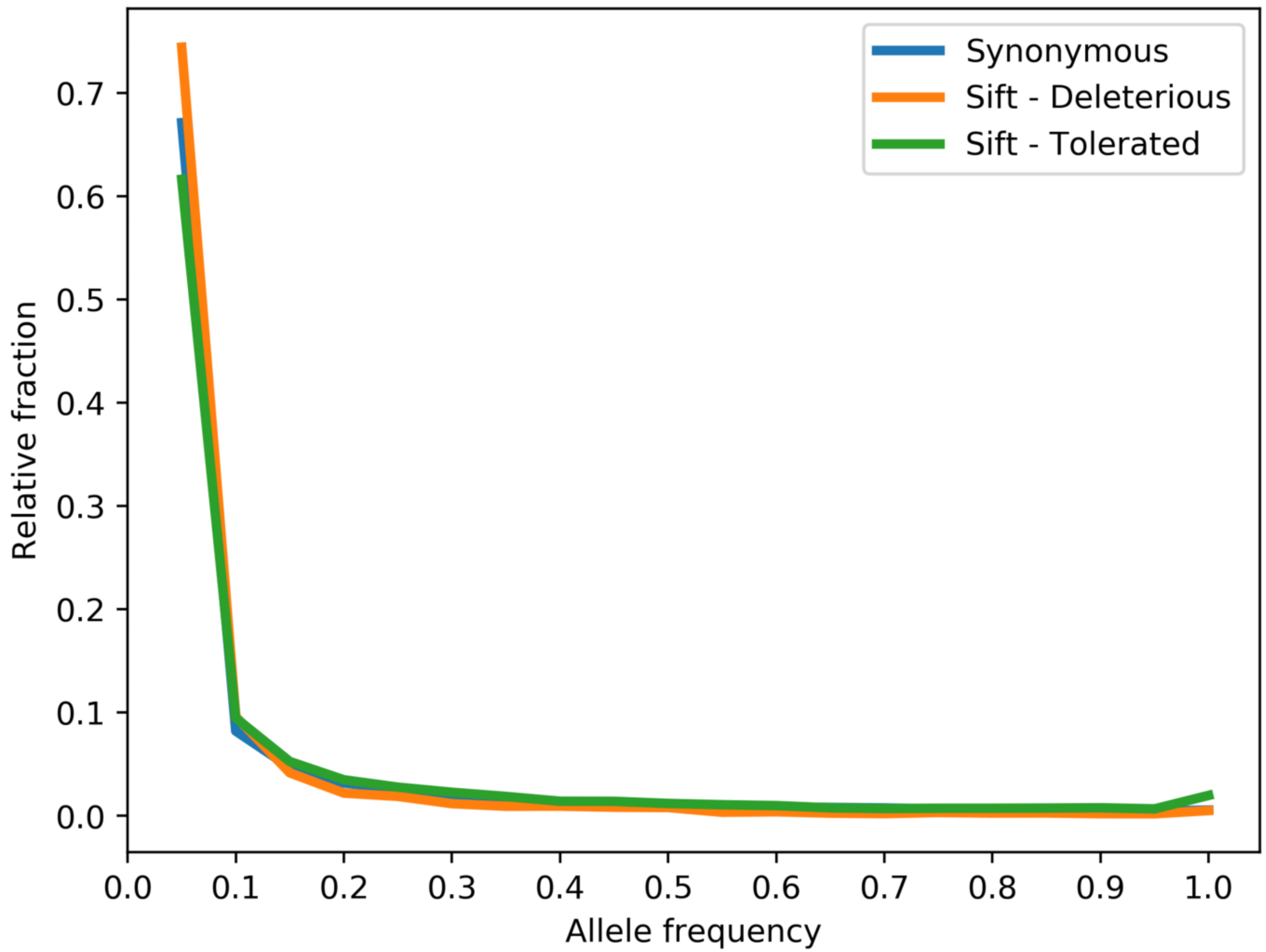

Figure S6: Allele frequency distribution for synonymous, tolerated, and deleterious missense variants. Deleterious alleles show a distinct allele frequency spectrum with generally a higher proportion of low-frequency alleles. This is shown by a higher relative fraction of deleterious variants in the lowest allele frequency bin, and relatively fewer fixed deleterious alleles.
