# Supplementary material for: Loss of function mutations in essential genes cause embryonic lethality in pigs
Source: PLoS Genet. 2019 Mar 15;15(3):e1008055. doi: 10.1371/journal.pgen.1008055 (PMC6436757; doi:10.1371/journal.pgen.1008055)
Supplement: S7 Fig — A) Indel length distribution for non-coding regions. B) Indel length distributions for coding regions. (PDF) [file pgen.1008055.s007.pdf]

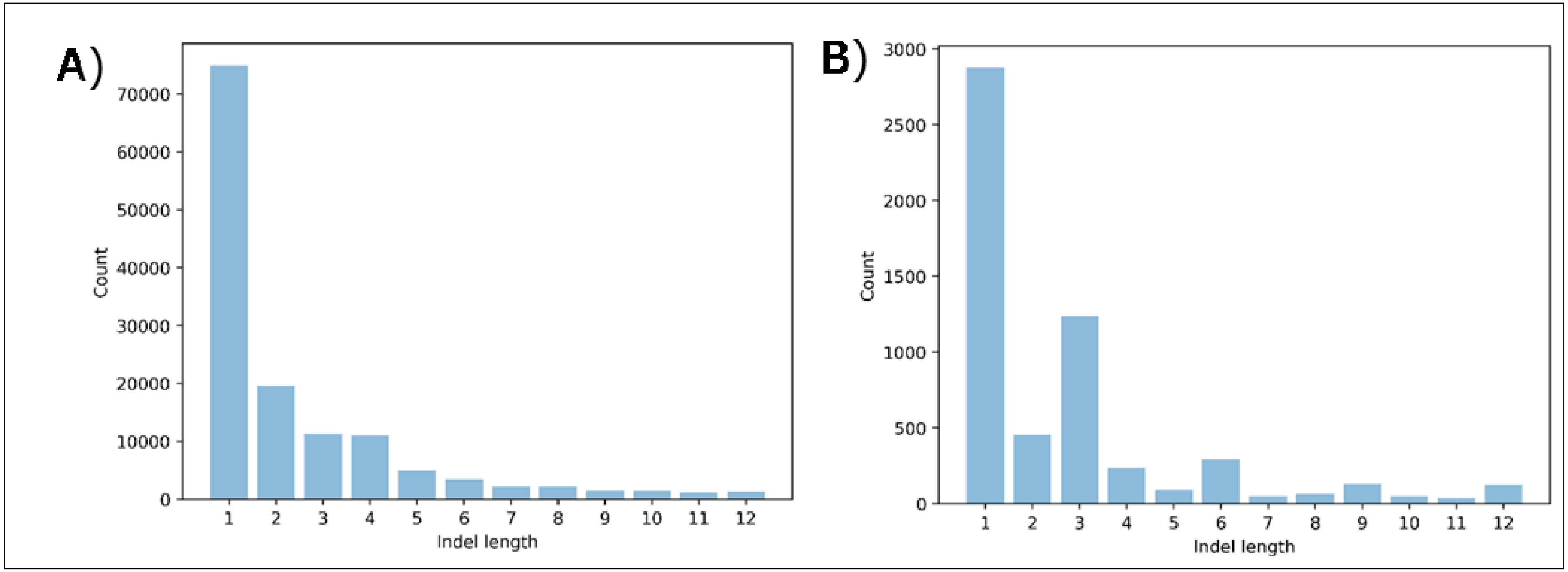

Figure S7: Enrichment of inframe indels in coding regions. A) Indel length distribution for non-coding regions. B) Indel length distributions for coding regions. Figure shows an enrichment of inframe indels in coding regions (e.g. length 3,6,9), not shown in non-coding regions.
