# Supplementary material for: Loss of function mutations in essential genes cause embryonic lethality in pigs
Source: PLoS Genet. 2019 Mar 15;15(3):e1008055. doi: 10.1371/journal.pgen.1008055 (PMC6436757; doi:10.1371/journal.pgen.1008055)
Supplement: S8 Fig — (PDF) [file pgen.1008055.s008.pdf]

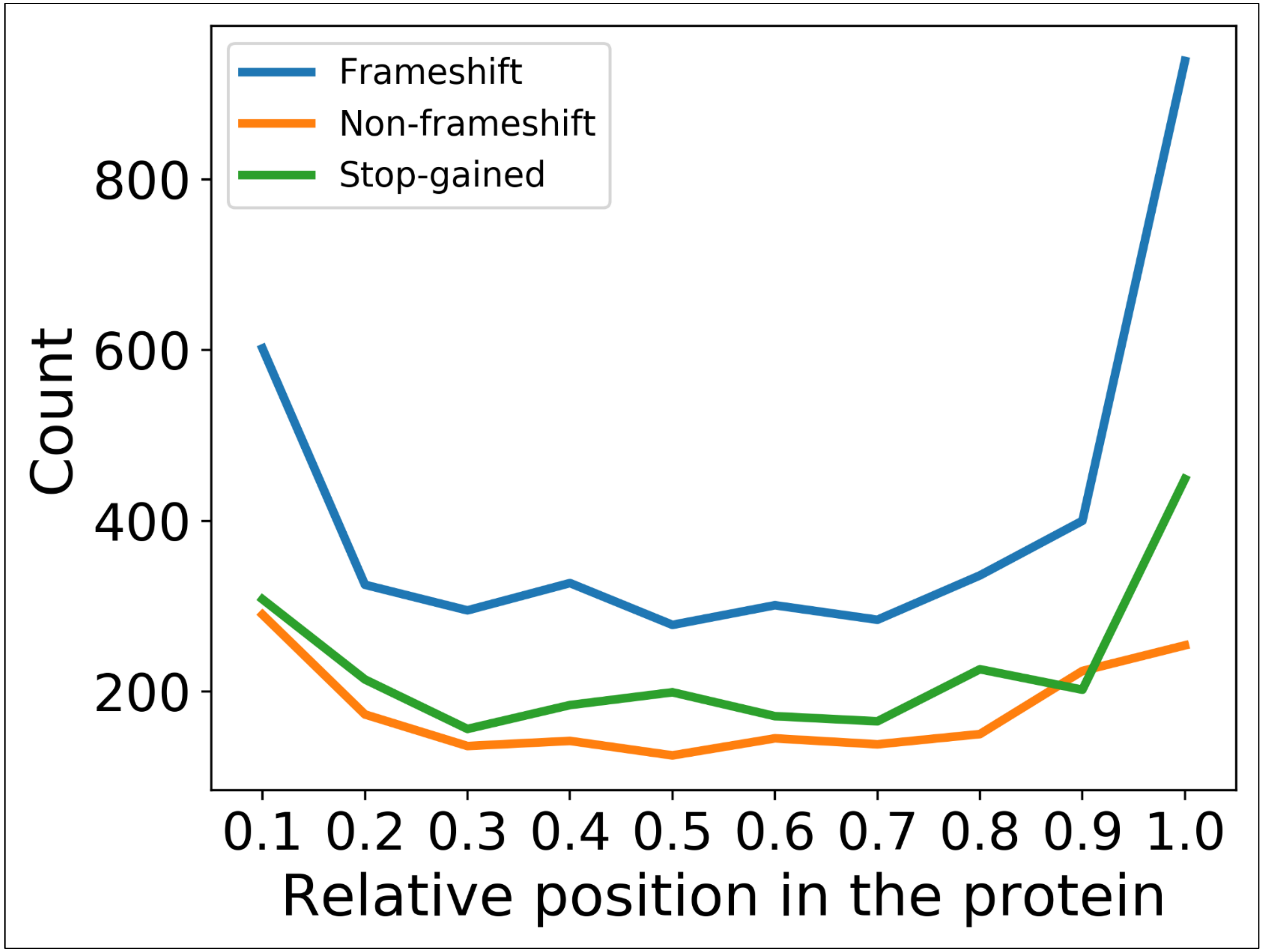

Figure S8: Relative position in the protein for frameshift, non-frameshift, and stop-gained variants in the pig populations. Frameshift variants are enriched in N- and C-terminal parts of the protein. Frameshift variants at the N-terminal sites are potentially "rescued" by alternate start-codons. Frameshift variants at the C-terminal end are less likely to be disruptive, since a functional protein might still be translated.
