# Supplementary material for: Loss of function mutations in essential genes cause embryonic lethality in pigs
Source: PLoS Genet. 2019 Mar 15;15(3):e1008055. doi: 10.1371/journal.pgen.1008055 (PMC6436757; doi:10.1371/journal.pgen.1008055)
Supplement: S9 Fig — (PDF) [file pgen.1008055.s009.pdf]

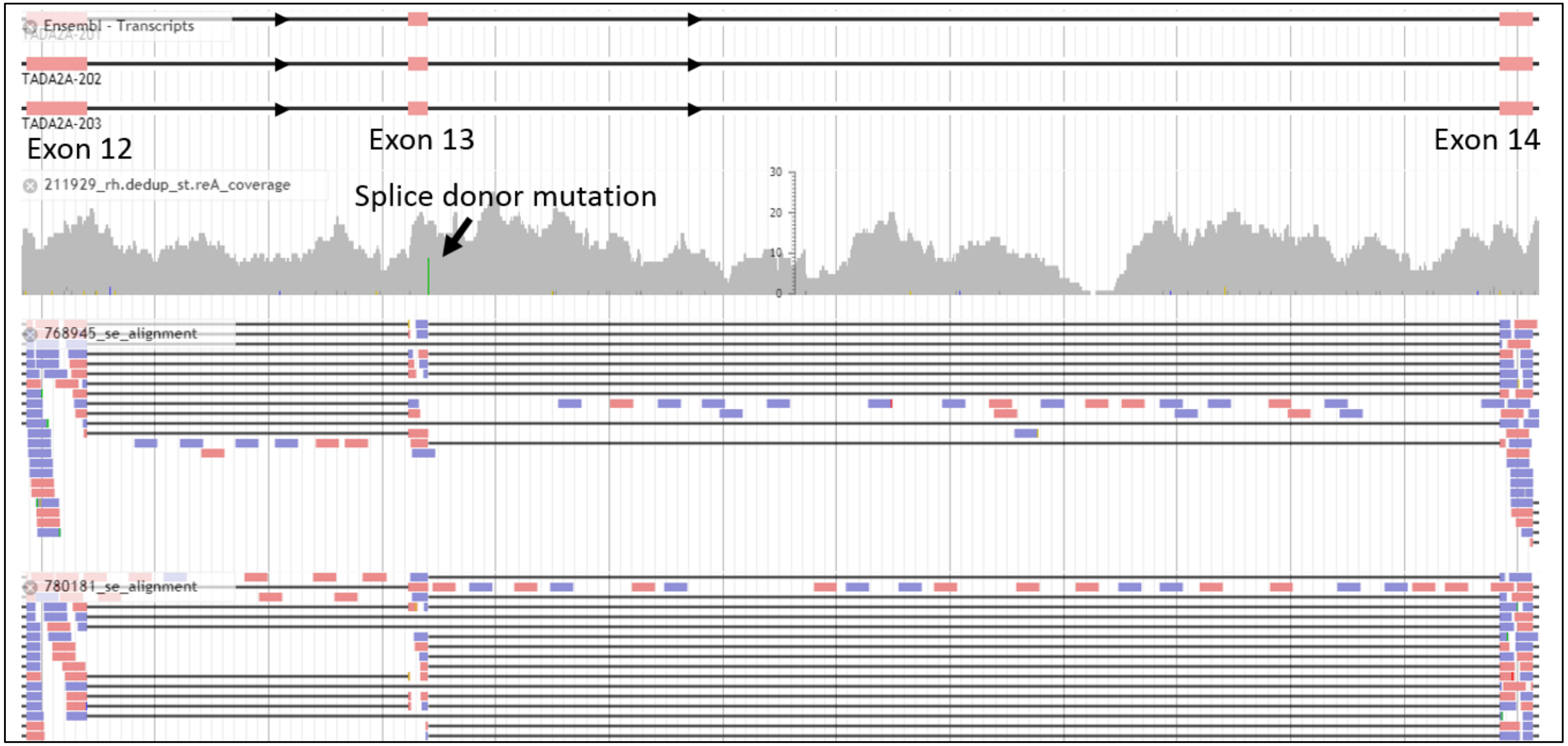

Figure S9: Screen capture shows exon skipping of *TADA2A* exon 13 in two carriers (768945, 780181) of the DU1 splice-donor mutation. Figure shows three Ensembl-predicted *TADA2A* transcripts on the forward strand and the coverage track of one carrier animal (L929) including the splice-donor mutation indicated with an arrow. The RNA-seq alignments show that both carrier samples exhibit four reads that splice directly from exon 12 to exon 14, not observed for non-carrier animals.
