# Supplementary material for: Loss of function mutations in essential genes cause embryonic lethality in pigs
Source: PLoS Genet. 2019 Mar 15;15(3):e1008055. doi: 10.1371/journal.pgen.1008055 (PMC6436757; doi:10.1371/journal.pgen.1008055)
Supplement: S10 Fig — (PDF) [file pgen.1008055.s010.pdf]

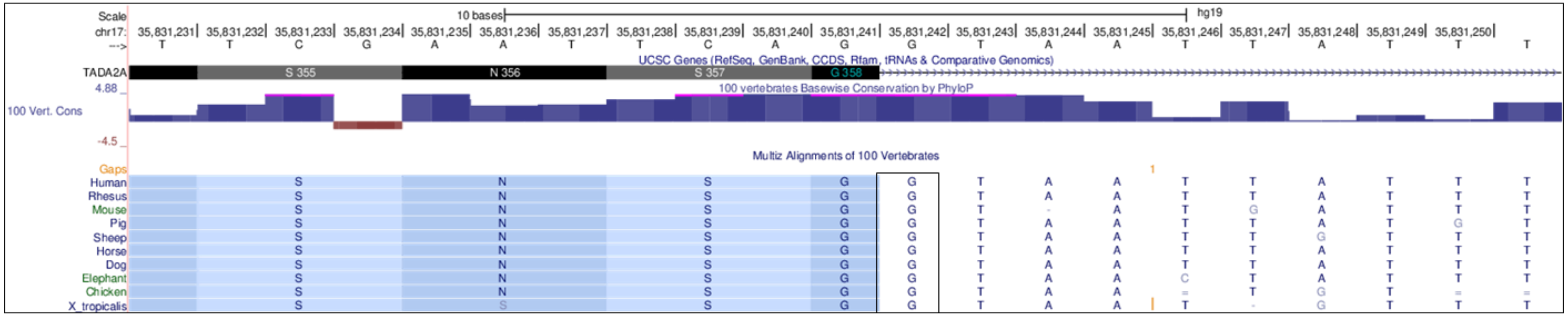

Figure S10: UCSC screen capture showing sequence conservation for the G̲T splice dinucleotide site in the *TADA2A* gene. Figure shows the human homologous sequence of the splice-donor site including the alignment of 8 mammalian, and two non-mammalian species (chicken and frog). The track '100 Vert. Cons' shows the basewise PhyloP sequence conservation scores. Figure shows complete conservation of the affected *TADA2A* splice-donor site region in all 10 species. The *TADA2A* affected splice-donor position is indicated in the black box.
