# Supplementary material for: Loss of function mutations in essential genes cause embryonic lethality in pigs
Source: PLoS Genet. 2019 Mar 15;15(3):e1008055. doi: 10.1371/journal.pgen.1008055 (PMC6436757; doi:10.1371/journal.pgen.1008055)
Supplement: S11 Fig — Screen capture of intron retention for one DU1 carrier sample (906564) at the 5’ (A), and 3’ end (B) of TADA2A intron 13 caused by the DU1 splice-donor mutation. (PDF) [file pgen.1008055.s011.pdf]

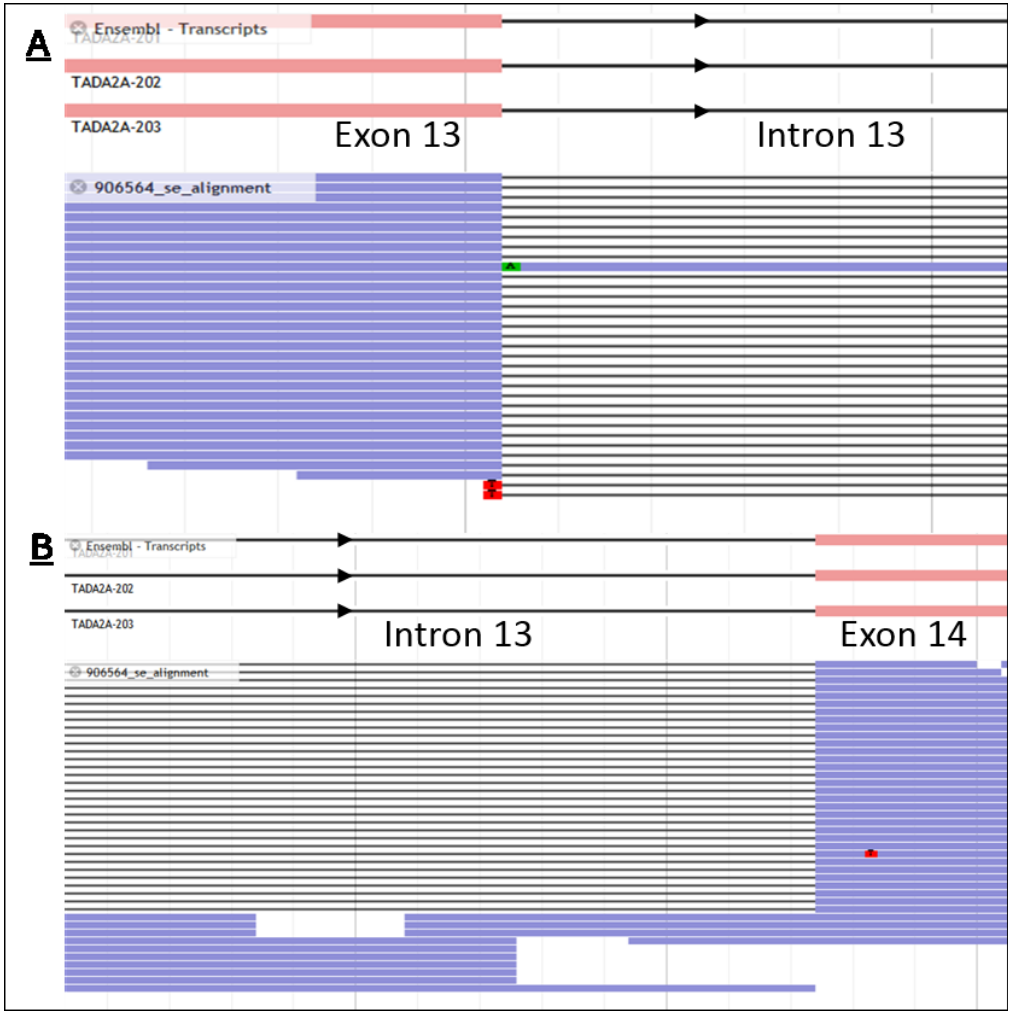

Figure S11: Screen capture of intron retention for one DU1 carrier sample (906564) at the 5' (A), and 3' end (B) of *TADA2A* intron 13 caused by the DU1 splice-donor mutation. Figure shows three Ensembl-predicted *TADA2A* transcripts on the forward strand and the alignment track of one carrier animal (906564). A). Exon 13-intron 13 splice region showing one read supporting intron retention. B). Exon 14-intron 13 splice region showing four read supporting intron retention.
