# Supplementary material for: Loss of function mutations in essential genes cause embryonic lethality in pigs
Source: PLoS Genet. 2019 Mar 15;15(3):e1008055. doi: 10.1371/journal.pgen.1008055 (PMC6436757; doi:10.1371/journal.pgen.1008055)
Supplement: S12 Fig — (PDF) [file pgen.1008055.s012.pdf]

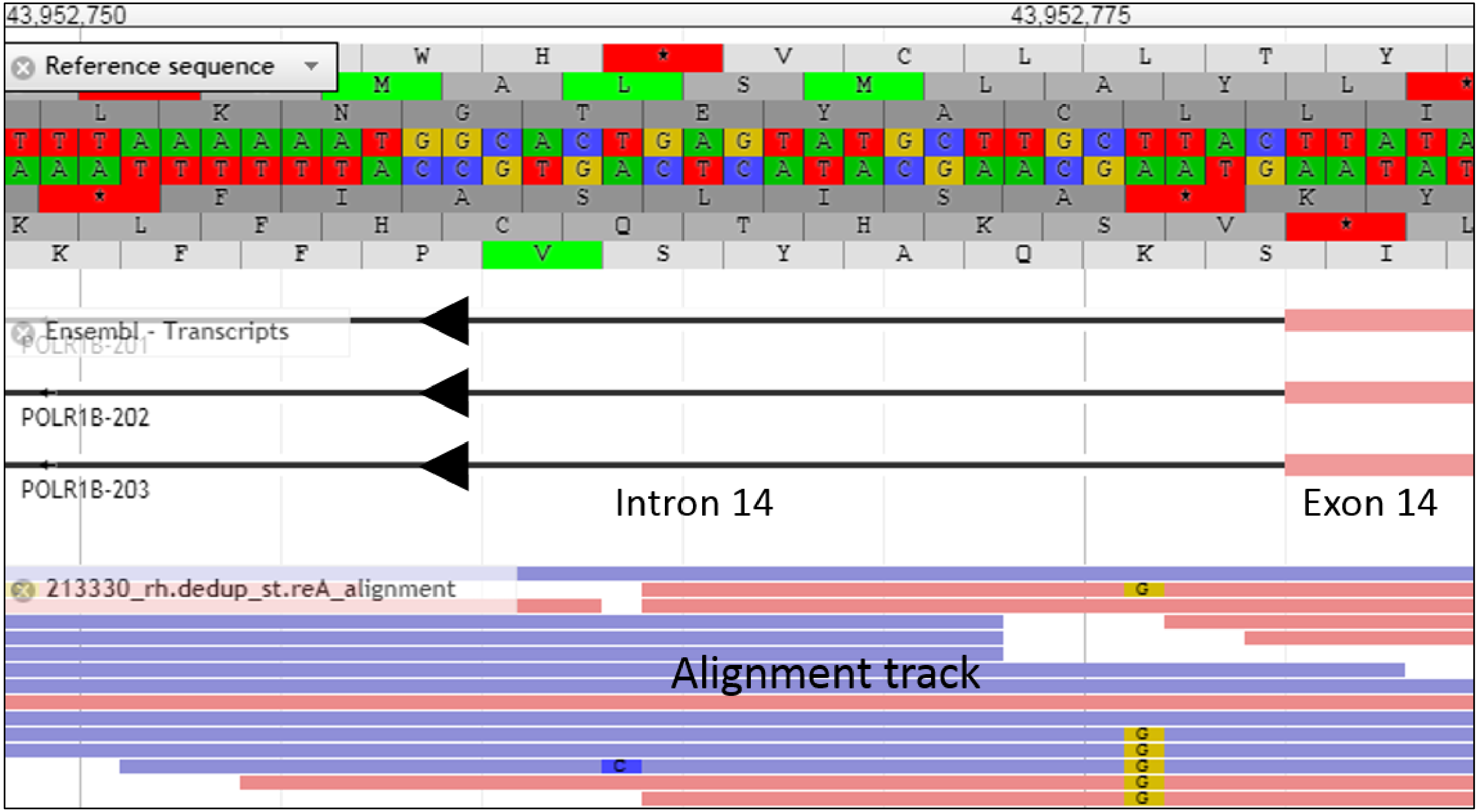

Figure S12: Screen capture showing the splice region mutation in intron 14 of the *POLR1B* gene in one of the LA1 carrier animals (L330). Figure shows the reference sequence for the tail of exon 14 and the start of intron 14 of *POLR1B* and three Ensembl-predicted *POLR1B* transcripts on the negative strand. The alignment track shows the all aligned reads and variants per position. The splice mutation affects a conserved adenine in the GTRAG splice site motif on the negative strand.
