# Supplementary material for: Loss of function mutations in essential genes cause embryonic lethality in pigs
Source: PLoS Genet. 2019 Mar 15;15(3):e1008055. doi: 10.1371/journal.pgen.1008055 (PMC6436757; doi:10.1371/journal.pgen.1008055)
Supplement: S13 Fig — A) UCSC screen capture of POLR1B splice region. B) Exon skipping of POLR1B exon 14 in one LA1 carrier animal. (PDF) [file pgen.1008055.s013.pdf]

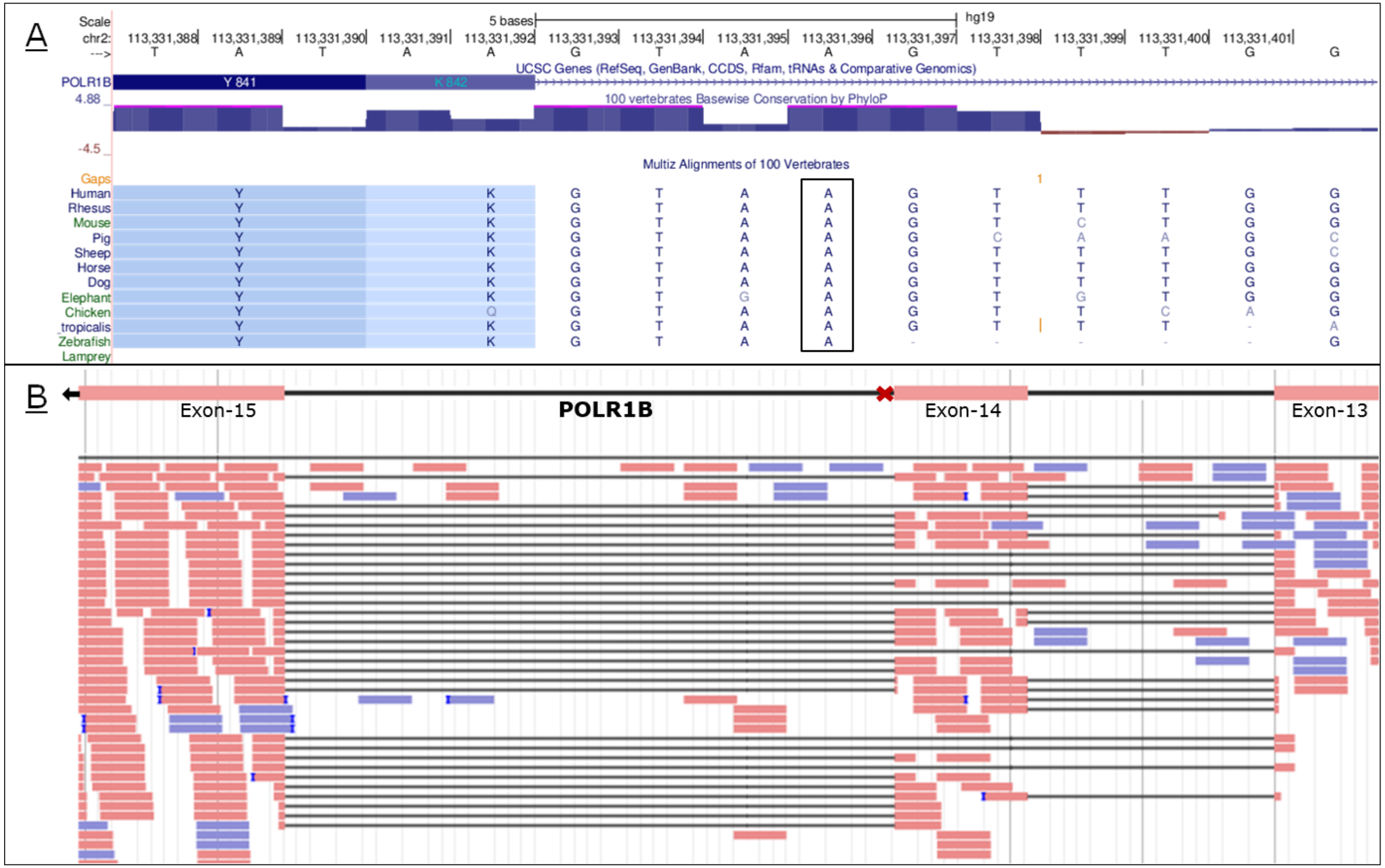

Figure S13: A) UCSC screen capture of *POLR1B* splice region. Figure shows the human homologous sequence of the splice-region site including the alignment of 8 mammalian, and three non-mammalian species (chicken, frog, and zebrafish). The track '100 Vert. Cons' shows the basewise PhyloP sequence conservation scores. The *POLR1B* affected splice-donor position is indicated in the black box. The GTRAG site affected by the LA1 splice-region mutation is strongly conserved in vertebrates in all 11 species. **B) Exon skipping of *POLR1B* exon 14 in one LA1 carrier animal**. Figure shows the Ensembl-predicted *POLR1B* transcript and the RNA-seq alignment from one carrier animal from exon 13 to exon 15.The location of the mutation is indicated with a red cross.
