# Supplementary material for: Loss of function mutations in essential genes cause embryonic lethality in pigs
Source: PLoS Genet. 2019 Mar 15;15(3):e1008055. doi: 10.1371/journal.pgen.1008055 (PMC6436757; doi:10.1371/journal.pgen.1008055)
Supplement: S14 Fig — (PDF) [file pgen.1008055.s014.pdf]

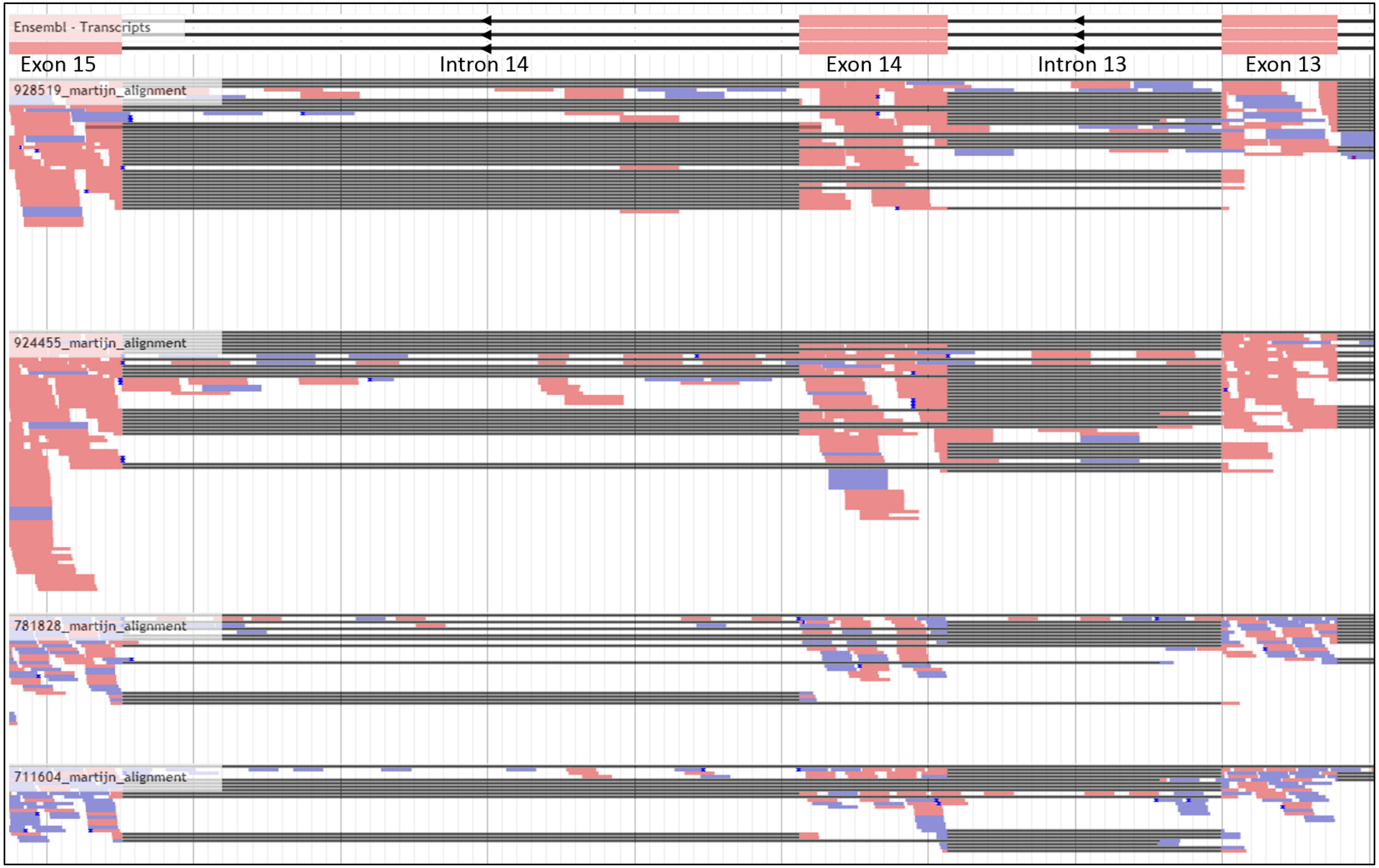

Figure S14: Screen capture of exon skipping (indicated by grey lines) in the *POLR1B* gene (exon 14) caused by the 3:g.43952776T>G splice region mutation. Figure shows three Ensembl-predicted *POLR1B* transcripts on the reverse strand and the alignment track of four carrier animals (928519, 924455, 781828. 711604).
