# Supplementary material for: Loss of function mutations in essential genes cause embryonic lethality in pigs
Source: PLoS Genet. 2019 Mar 15;15(3):e1008055. doi: 10.1371/journal.pgen.1008055 (PMC6436757; doi:10.1371/journal.pgen.1008055)
Supplement: S15 Fig — (PDF) [file pgen.1008055.s015.pdf]

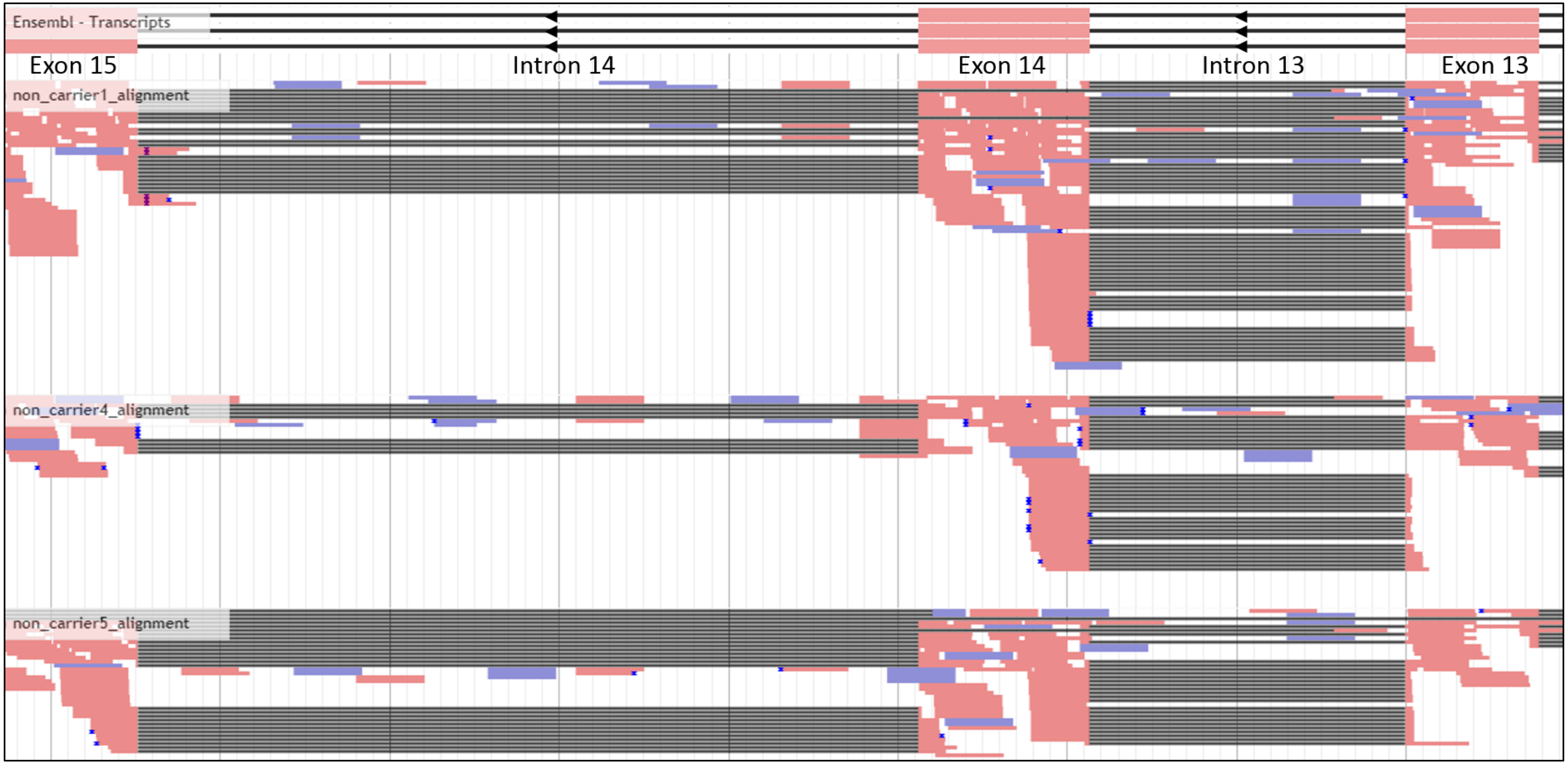

Figure S15: Screen capture of three non-carrier animals of the LA1 splice-region mutation. Figure shows three Ensembl-predicted *POLR1B* transcripts on the reverse strand and the alignment track of three non-carrier animals. Non-carriers show no exon-skipping of *POLR1B* exon 14.
