# Supplementary material for: Loss of function mutations in essential genes cause embryonic lethality in pigs
Source: PLoS Genet. 2019 Mar 15;15(3):e1008055. doi: 10.1371/journal.pgen.1008055 (PMC6436757; doi:10.1371/journal.pgen.1008055)
Supplement: S16 Fig — (PDF) [file pgen.1008055.s016.pdf]

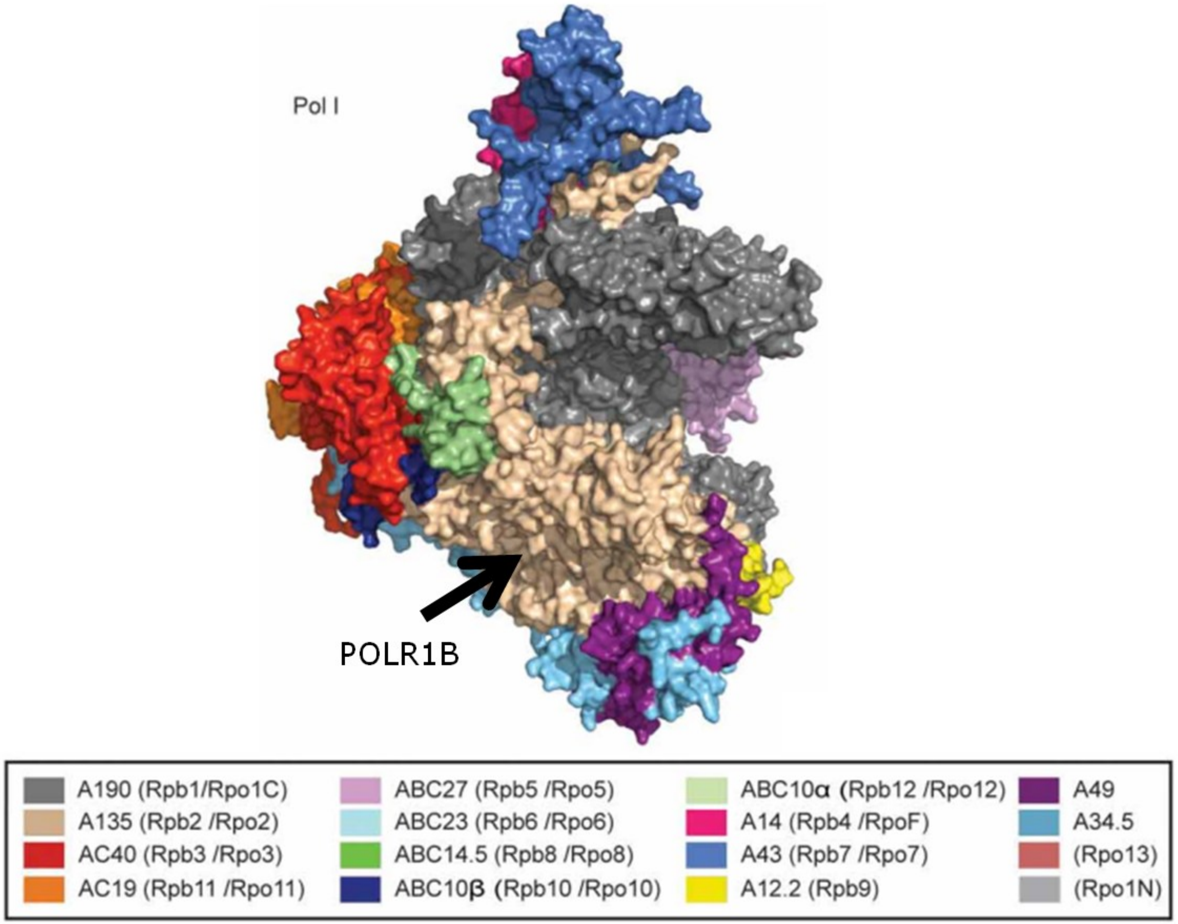

Figure S16: Protein structure of RNA-polymerase 1. Figure shows all subunits including the A135 subunit encoded by the LA1 affected *POLR1B* gene (indicated with an arrow). Figure adjusted from Fernandez-Tornero et al. 2013 (Fernandez-Tornero et al. 2013).

Fernandez-Tornero C, Moreno-Morcillo M, Rashid UJ, Taylor NM, Ruiz FM, Gruene T, Legrand P, Steuerwald U, Muller CW. 2013. Crystal structure of the 14-subunit RNA polymerase I. *Nature* **502**: 644-649.
