# Supplementary material for: Loss of function mutations in essential genes cause embryonic lethality in pigs
Source: PLoS Genet. 2019 Mar 15;15(3):e1008055. doi: 10.1371/journal.pgen.1008055 (PMC6436757; doi:10.1371/journal.pgen.1008055)
Supplement: S17 Fig — (PDF) [file pgen.1008055.s017.pdf]

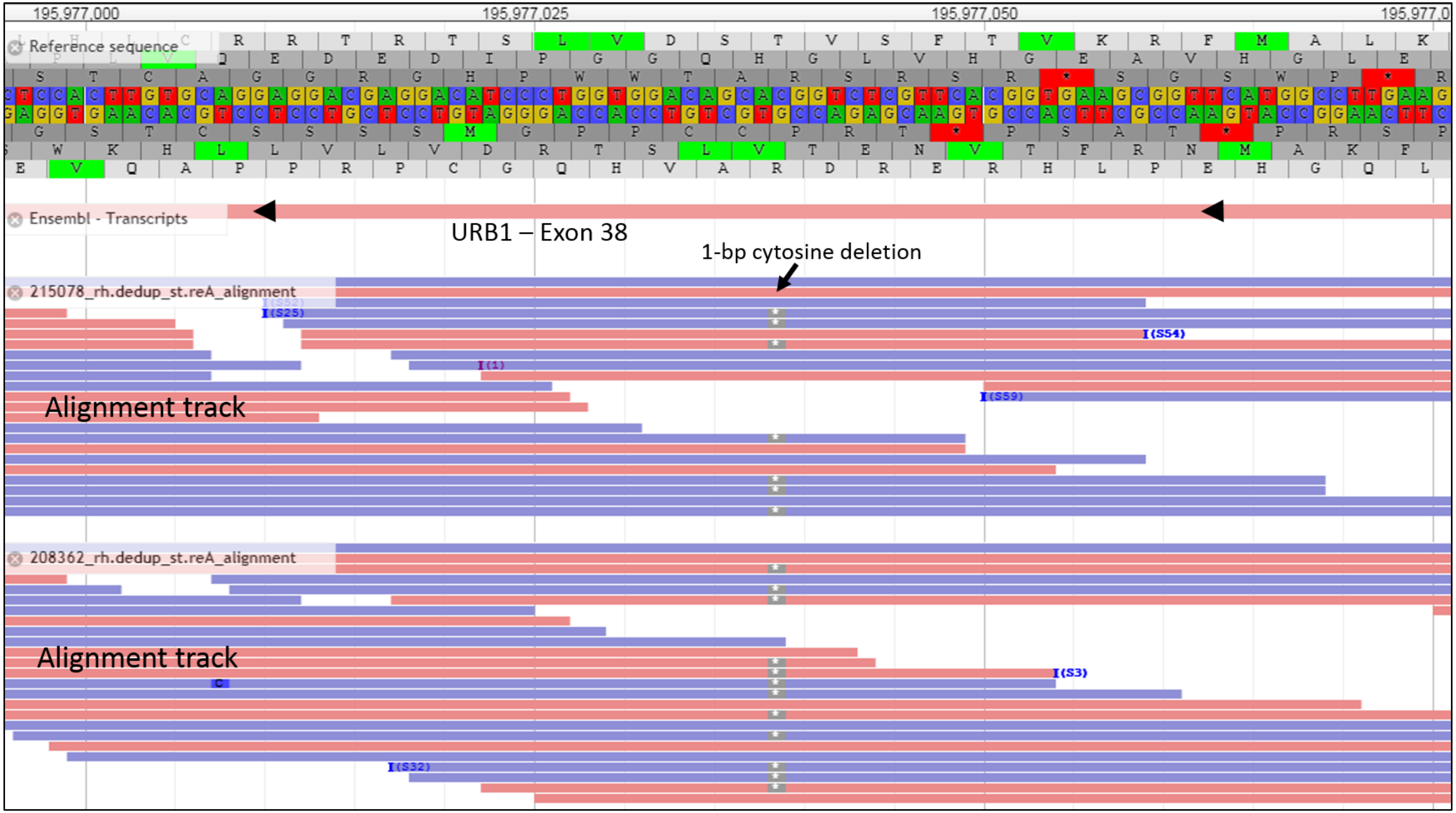

Figure S17: Screen capture of two carrier animals (L078, L362) for the LA2 (p.Val1961fs) frameshift mutation. Figure shows the single Ensembl-predicted URB1 transcript on the reverse strand and the alignment track of two LA2 carrier animals. The 1 basepair deletion in the coding region of exon 38 in the *URB1* gene is indicated with an arrow.
