# Supplementary material for: Loss of function mutations in essential genes cause embryonic lethality in pigs
Source: PLoS Genet. 2019 Mar 15;15(3):e1008055. doi: 10.1371/journal.pgen.1008055 (PMC6436757; doi:10.1371/journal.pgen.1008055)
Supplement: S18 Fig — (PDF) [file pgen.1008055.s018.pdf]

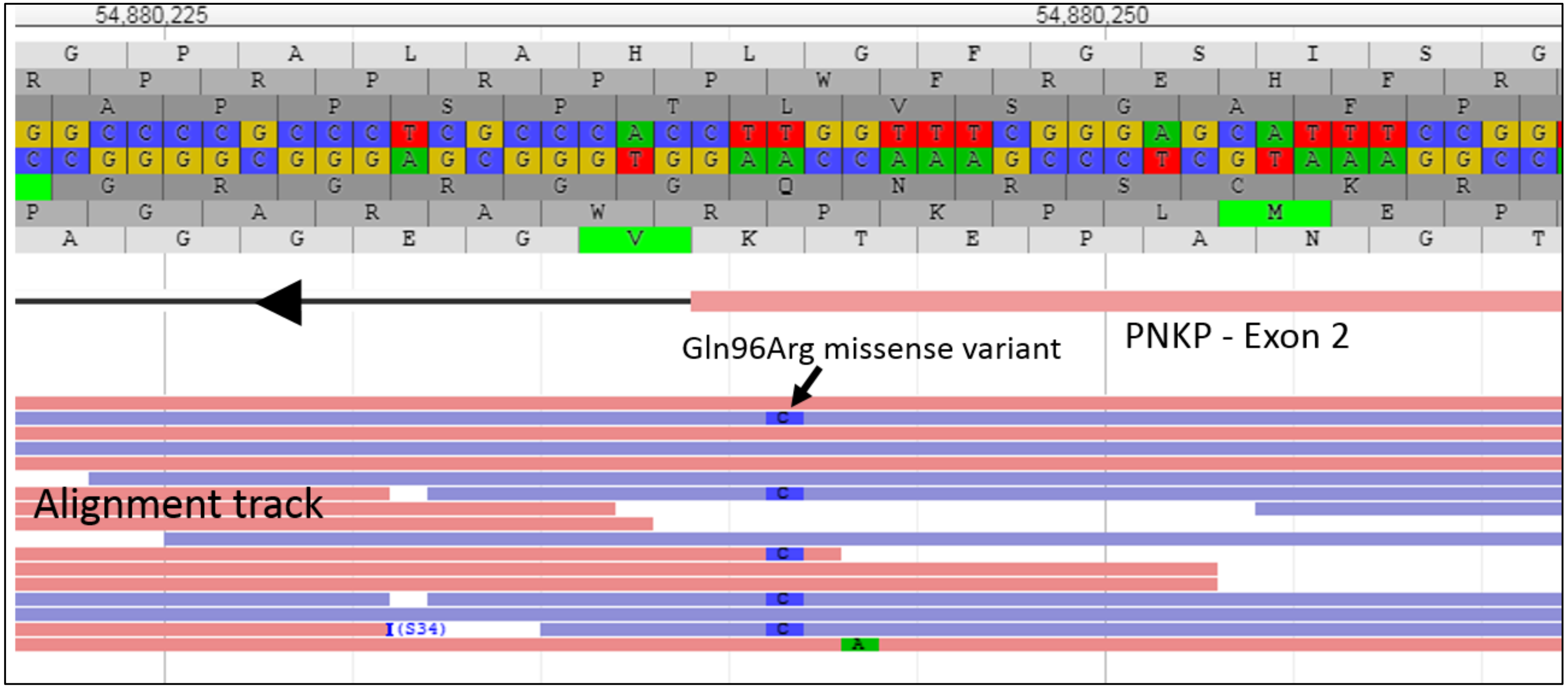

Figure S18: Screen capture of one carrier animals (L827) for the LA3 missense mutation. Figure shows the tail of *PNKP* exon 2 including the 6:g.54880241G>T substitution resulting in a deleterious ENSSSCP00000003467:p.Gln96Arg substitution in the PNKP protein.
