# Supplementary material for: Loss of function mutations in essential genes cause embryonic lethality in pigs
Source: PLoS Genet. 2019 Mar 15;15(3):e1008055. doi: 10.1371/journal.pgen.1008055 (PMC6436757; doi:10.1371/journal.pgen.1008055)
Supplement: S19 Fig — (PDF) [file pgen.1008055.s019.pdf]

```
Pig_WT          LESPPGGAPPIFLPSDGQALVLGRGPLTLVTDRKCSRNQVELVADPETRTVAVKQLGVNP
Pig_Mutant      LESPPGGAPPIFLPSDGQALVLGRGPLTLVTDRKCSRNRVELVADPETRTVAVKQLGVNP
Human           LESPPGGAPPIFLPSDGQALVLGRGPLTQVTDRKCSRTQVELVADPETRTVAVKQLGVNP
Chimpanzee      LESPPGGAPPIFLPSDGQALVLGRGPLTQVTDRKCSRTQVELVADPETRTVAVKQLGVNP
Dog             LESPPGGAPPIFLPSDGQALVLGRGPLTQVTDRKCSRNQVELVANPRTRTVAVKQLGINP
Cattle          LESPAGGAPPIFLPTGGQALVLGRGPLTQVTDRKCSRNQVELVADPETRTVAVKQLGVNP
Mouse           LQSPTGGPPPIFLPSDGQALVLGRGPLTQVTDRKCSRNQVELIADPESRTVAVKQLGVNP
Zebrafish       LVSVD---GDRVVLPDGRALMFGRGPESRISDKKCSRHQVKLVADYAKQEVLVTQLGPNP
Fruit_fly       LKPTEPE-HHSIHL-TAGENFVGRSRETGIRDSKCSKRQIQLQVDLKKAVVSLKVLGVNP
                *          .    .   ..**.  : : * ***: :::* .:  .  * :. ** **
```

Figure S19: PNKP multiple sequence alignment. Figure shows the protein alignment of 6 mammalian, and two non-mammalian species (zebrafish, fruitfly). The PNKP (ENSSSCP00000003467:p.Gln96Arg) missense position (indicated with a blue box) is fully conserved across a wide range of vertebrate species.
