# Supplementary material for: Loss of function mutations in essential genes cause embryonic lethality in pigs
Source: PLoS Genet. 2019 Mar 15;15(3):e1008055. doi: 10.1371/journal.pgen.1008055 (PMC6436757; doi:10.1371/journal.pgen.1008055)
Supplement: S1 Table — (PDF) [file pgen.1008055.s020.pdf]

Table S1: Number of genotypes and sequenced individuals. The number of trios are the number of genotyped animals that have both parents genotyped.

Breed	#80K genotyped	#Trio's	#Duo's	#WGS
Landrace	28,085	15,631	10,792	167
Duroc	11,255	3,497	7,498	119
