# Supplementary material for: Loss of function mutations in essential genes cause embryonic lethality in pigs
Source: PLoS Genet. 2019 Mar 15;15(3):e1008055. doi: 10.1371/journal.pgen.1008055 (PMC6436757; doi:10.1371/journal.pgen.1008055)
Supplement: S2 Table — (PDF) [file pgen.1008055.s021.pdf]

Table S2: Phenotype records for CxC and CxNC matings (TNB: total number born, NBA: number born alive, NSB: number stillborn, MUM: number mummified). The number of mummified piglets is not recorded on every farm, resulting in fewer phenotype records.

**Hap.**	**# CxC (TNB/NBA/NSB)**	**# CxC (MUM)**	**#CxNC (TNB/NBA/NSB)**	**#CxNC (MUM)**
LA1	297	106	2,350	778
LA2	127	49	1,527	513
LA3	30	11	872	294
LA4	29	5	950	300
DU1	21	1	293	37
