# Supplementary material for: Loss of function mutations in essential genes cause embryonic lethality in pigs
Source: PLoS Genet. 2019 Mar 15;15(3):e1008055. doi: 10.1371/journal.pgen.1008055 (PMC6436757; doi:10.1371/journal.pgen.1008055)
Supplement: S3 Table — (PDF) [file pgen.1008055.s022.pdf]

Table S3: Fertility phenotypes for liveborn, stillborn, and mummified piglets for CxC litters compared to CxNC litters. CxC litters show clear reduction in liveborn (12.1-23%). However, no increase in the number of stillbirths or the number mummified piglets is observed.

Haplotype	Phenotype	CxC mean	CxNC mean	Ratio	P-val
LA1	TOTALBORN	11.51	14.18	0.814	<0.000001
LA2	TOTALBORN	12.00	14.26	0.841	<0.000001
LA3	TOTALBORN	11.96	14.09	0.849	0.00064
LA4	TOTALBORN	11.48	14.05	0.817	0.0075
DU1	TOTALBORN	7.33	9.35	0.784	0.0028
LA1	LIVEBORN	10.67	12.93	0.825	<0.000001
LA2	LIVEBORN	11.19	13.04	0.858	<0.000001
LA3	LIVEBORN	11.37	12.92	0.880	0.0068
LA4	LIVEBORN	10.72	12.84	0.835	0.013
DU1	LIVEBORN	6.62	8.54	0.775	0.006
LA1	STILLBORN	0.85	1.26	0.675	<0.000001
LA2	STILLBORN	0.81	1.23	0.859	0.007
LA3	STILLBORN	0.60	1.15	0.522	0.11
LA4	STILLBORN	0.76	1.21	0.628	0.11
DU1	STILLBORN	0.80	0.91	0.879	0.65
LA1	MUMMIES	0.45	0.42	1.072	0.83
LA2	MUMMIES	0.18	0.42	0.437	0.0028
LA3	MUMMIES	0.09	0.48	0.19	0.0021
LA4	MUMMIES	0.2	0.39	0.517	0.41
DU1	MUMMIES	0	0.162	0.000	-
