# Supplementary material for: Loss of function mutations in essential genes cause embryonic lethality in pigs
Source: PLoS Genet. 2019 Mar 15;15(3):e1008055. doi: 10.1371/journal.pgen.1008055 (PMC6436757; doi:10.1371/journal.pgen.1008055)
Supplement: S4 Table — (PDF) [file pgen.1008055.s023.pdf]

Table S4: WGS carrier animals for candidate lethal haplotypes. Table shows the carrier IDs of all identified carriers of the recessive lethal haplotypes.

Hap.	# WGS carriers	Carrier identifiers
LA1	21	L528,L051,L134,L138,L330,L156,L167,L179,L181,L191,L196,L197,L891,L911,L957,L958, L972,L976,L988,L991,L029
LA2	17	L528,L965,L362,L078,L147,L151,L163,L174,L175,L200,L892,L898,L921,L991,L006,L008,L049
LA3	7	L175,L185,L827,L844,L936,L944,L002
LA4	9	L330,L156,L067,L197,L202,L911,L970,L972,L024
DU1	9	D833, D929, D917,D929,D948,D964,D981,D035,D416
