# Supplementary material for: Loss of function mutations in essential genes cause embryonic lethality in pigs
Source: PLoS Genet. 2019 Mar 15;15(3):e1008055. doi: 10.1371/journal.pgen.1008055 (PMC6436757; doi:10.1371/journal.pgen.1008055)
Supplement: S5 Table — (PDF) [file pgen.1008055.s024.pdf]

Table S5: Per-haplotype RNA-seq carrier animals. Table shows the birth date and sequenced tissue for each carrier animal. No RNA-seq data for LA3 or LA4 carriers was available.

Haplotype	Carrier identifiers	Birth date	Tissue
LA1	711604	29.08.2010	Testis
LA1	781828	20.11.2011	Testis
LA1	924455	05.05.2014	Testis
LA1	928519	05.05.2014	Testis
LA2	880613	09.07.2013	Testis
LA2	883315	03.08.2013	Testis
LA2	906554	17.12.2013	Testis
DU1	768945	07.09.2011	Testis
DU1	780181	16.11.2011	Testis
DU1	906564	13.12.2013	Testis
