# Supplementary material for: Loss of function mutations in essential genes cause embryonic lethality in pigs
Source: PLoS Genet. 2019 Mar 15;15(3):e1008055. doi: 10.1371/journal.pgen.1008055 (PMC6436757; doi:10.1371/journal.pgen.1008055)
Supplement: S7 Table — (PDF) [file pgen.1008055.s026.pdf]

Table S7: Wildtype and LA1 and recombinant haplotype in the region SSC3: 42.5-47.5. The LA1 recessive lethal haplotype is indicated in green, while the wild-type haplotype is indicated in yellow. The Wildtype - LA1 recombinant haplotype is homozygous for the LA1 haplotype in 45.6-47.5, excluding this region to carry the causal mutation.

Marker	Wildtype	LA1	Wildtype - LA1 recombinant
42638945_GA	**G**	G	**G**
42838594_AG	**A**	A	**A**
43055325_AG	**A**	A	**A**
43167191_AG	**A**	A	**A**
43199265_GA	**G**	G	**G**
43227069_AG	**A**	A	**A**
43312168_AG	**A**	A	**A**
43463318_AG	**A**	A	**A**
43561730_AG	**A**	A	**A**
44000093_AG	**A**	A	**A**
44184377_GA	**G**	G	**G**
44449854_GA	**G**	G	**G**
44807671_AC	**A**	C	**A**
44982953_CA	**C**	C	**C**
45234651_GA	**G**	G	**G**
45458899_AG	**A**	G	**A**
45482426_GA	**G**	A	**G**
45505509_GA	**G**	A	**G**
45593674_AG	**A**	G	**A**
45625955_GA	**G**	A	**G**
45825165_AG	A	G	G
45844527_GA	G	A	A
45940448_AG	A	A	A
46017895_GA	G	G	G
46032503_AG	A	A	A
46058043_GA	G	G	G
46125819_AG	A	A	A
46297805_AG	A	G	G
46321828_AG	A	A	A
46387029_AG	A	A	A
46398474_AG	A	A	A
46520543_AC	A	C	C
46617035_CG	C	C	C
46622746_AG	A	A	A
46641455_AC	A	A	A
46708328_AG	A	A	A
46710098_AG	A	A	A
46716351_AC	A	C	C
46864677_GA	G	A	A
46908317_AG	A	G	G
46945304_AG	A	A	A
46962784_GA	G	G	G
46995328_GA	G	A	A
47160410_CA	C	A	A
47174278_GA	G	A	A
47187516_AG	A	A	A
47214285_AG	A	A	A
47274629_AG	A	A	A
47337139_GA	G	G	G
47357791_GA	G	A	A
47380153_GA	G	A	A
47393273_CA	C	C	C
47417411_AC	A	C	C
47473518_CA	C	C	C
47489076_GA	G	G	G
