# Supplementary material for: Loss of function mutations in essential genes cause embryonic lethality in pigs
Source: PLoS Genet. 2019 Mar 15;15(3):e1008055. doi: 10.1371/journal.pgen.1008055 (PMC6436757; doi:10.1371/journal.pgen.1008055)
Supplement: S8 Table — (PDF) [file pgen.1008055.s027.pdf]

Table S8: Two recombinant samples used for fine-mapping of the LA1 haplotype. The LA1 recessive lethal haplotype is indicated in green, while the wild-type haplotype is indicated in yellow. Both animals are homozygous for part of the LA1 haplotype.

Pig ID	Haplotype 1	Haplotype 2
3582	GAAAGAAAAAGGACGAGGAGAGAGAGAGAAACCAAAACAGAGAAAAAAGAACCCG	GAAAGAAAAAGGCCGGAAGAGAAGAGAGAAACCAAAACAGAGAAAAAAGAACCCG
8305	GAAAGAAAAAGGCCGGAAGAGAAGAGAGAAACCAAAACAGAGAAAAAAGAACCCG	GAAAGAAAAAGGACGAGGAGGAAGAGAGAAACCAAAACAGAGAAAAAAGAACCCG
