# Supplementary material for: Loss of function mutations in essential genes cause embryonic lethality in pigs
Source: PLoS Genet. 2019 Mar 15;15(3):e1008055. doi: 10.1371/journal.pgen.1008055 (PMC6436757; doi:10.1371/journal.pgen.1008055)
Supplement: S12 Table — (PDF) [file pgen.1008055.s031.pdf]

Table S12: RNA-seq expression in fragments per kilobase per million (FPKM) for wild-type and mutant transcript. Table shows the FPKM expression values for both the wild-type (Wt) and mutant (exon-skipping) transcript.

**Hap.**	**Carrier identifiers**	**Gene**	**Transcript IDs (Effect)**	**Wild-type**	**Mutant**
LA1	711604	*POLR1B*	TCONS_35434 (Wt), TCONS_35427 (exon skipping)	627.35	126.24
LA1	781828	*POLR1B*	TCONS_35434 (Wt), TCONS_35427 (exon skipping)	568.95	136.61
LA1	924455	*POLR1B*	TCONS_35434 (Wt), TCONS_35427 (exon skipping)	483.44	99.21
LA1	928519	*POLR1B*	TCONS_35434 (Wt), TCONS_35427 (exon skipping)	451.92	128.61
DU1	768945	*TADA2A*	TCONS_9105 (Wt), TCON_9101(intron retention), TCONS_9107 (exon skipping)	495.56	39.33, 195.53
DU1	780181	*TADA2A*	TCONS_9105 (Wt), TCON_9101(intron retention), TCONS_9107 (exon skipping)	517.72	61.84, 159.46
DU1	906564	*TADA2A*	TCONS_9105 (Wt), TCON_9101(intron retention), TCONS_9107 (exon skipping)	310.01	83.59, 269.6
