# Supplementary material for: Loss of function mutations in essential genes cause embryonic lethality in pigs
Source: PLoS Genet. 2019 Mar 15;15(3):e1008055. doi: 10.1371/journal.pgen.1008055 (PMC6436757; doi:10.1371/journal.pgen.1008055)
Supplement: S13 Table — (PDF) [file pgen.1008055.s032.pdf]

Table S13: Validation of LA1 causal mutation in three carrier-by-carrier litters. Sow (red) and Boar (blue) and complete liveborn (male and female) and stillborn progeny are genotyped for the T-43952776-G candidate causal mutation. No homozygous GG genotypes, and an excess of heterozygous GT genotypes are observed.

**Litter 1 / IDs**	**Genotype**	**Sex**		**Litter 2 / IDs**	**Genotype**	**Sex**		**Litter 3 / IDs**	**Genotype**	**Sex**
L151	GT	Sow		L707	GT	Sow		L038	GT	Sow
L207	GT	Boar		L207	GT	Boar		L006	GT	Boar
P489	TT	Male		P430	GT	Male		P276	TT	Male
P490	GT	Male		P433	GT	Male		P935	GT	Female
P965	GT	Female		P428	GT	Female		P933	GT	Female
P964	GT	Female		P429	GT	Female		P275	TT	Male
P487	TT	Male		P425	GT	Female		P273	GT	Male
P488	GT	Male		P424	GT	Female		P272	TT	Male
P966	GT	Female		P431	GT	Male		P936	GT	Female
P963	GT	Female		P423	TT	Female		P937	TT	Female
P962	GT	Female		P432	TT	Male		P938	GT	Female
P485	TT	Female		P426	GT	Female		P271	TT	Male
P967	TT	Female		P427	TT	Female		P274	TT	Male
P486	GT	Male						P934	TT	Female
P491	GT	Male						P542	GT	Stillborn
P553	GT	Stillborn								
**Wt=TT**	**Het=GT**	**Lethal=GG**		**Wt=TT**	**Het=GT**	**Lethal=GG**		**Wt=TT**	**Het=GT**	**Lethal=GG**
4	10	0		3	8	0		7	6	0
