# Supplementary material for: Loss of function mutations in essential genes cause embryonic lethality in pigs
Source: PLoS Genet. 2019 Mar 15;15(3):e1008055. doi: 10.1371/journal.pgen.1008055 (PMC6436757; doi:10.1371/journal.pgen.1008055)
Supplement: S14 Table — (PDF) [file pgen.1008055.s033.pdf]

Table S14: Validation of LA2 causal mutation in one carrier-by-carrier litters. Sow (red) and Boar (blue) and complete liveborn (male and female) and stillborn progeny are genotyped for the ACG-195977037-AG candidate causal frameshift deletion (1bp). No homozygous DEL/DEL genotypes, and an excess of heterozygous C/DEL genotypes are observed.

Animal ID	Genotype	Sex
L035	C/DEL	Sow
L192	C/DEL	Boar
P944	C/DEL	Female
P946	CC	Female
P278	CC	Male
P939	C/DEL	Female
P942	C/DEL	Female
P277	C/DEL	Male
P279	C/DEL	Male
P943	C/DEL	Female
P940	C/DEL	Female
P941	CC	Female
P945	C/DEL	Female
P540	C/DEL	Stillborn
P544	C/DEL	Stillborn
Wt=CC	Het=C/DEL	Lethal=DEL/DEL
3	10	0
