# Supplementary material for: Loss of function mutations in essential genes cause embryonic lethality in pigs
Source: PLoS Genet. 2019 Mar 15;15(3):e1008055. doi: 10.1371/journal.pgen.1008055 (PMC6436757; doi:10.1371/journal.pgen.1008055)
Supplement: S15 Table — (PDF) [file pgen.1008055.s034.pdf]

Table S15: Association analysis for LA1 carriers. AA represents the number of non-carriers in the database, while AB represents the number of carrier animals. The carriers of the haplotype LA1, compared to non-carriers, show: Increased carcass quality [+ loin depth (LDE), - backfat (BFE), + meat percentage (MTP)], Lower meat quality [- IMF, + drip loss (DRY)], slower growth (Days required to gain 40 kg), Increased mothering ability (MAB) [- dead at 21 days, + weight at 21 days].

Source	AA	AB	P value	-log10(P value)	effect	se	Trait
LA1	24479	3606	2.18E-41	41	0.356	0.026	Days required from 80-120 kg.
LA1	24475	3606	1.39E-34	34	0.306	0.025	Lean meat percentage by CT
LA1	24476	3606	3.53E-30	29	0.507	0.044	Maternal effect 21 days litter weight from reproduction analysis
LA1	24475	3606	3.99E-27	26	0.569	0.053	Days required from 40-120 kg
LA1	24476	3606	2.97E-22	22	0.258	0.027	Days required from 40-80 kg
LA1	24476	3606	2.28E-20	20	-0.013	0.001	Intramuscular fat
LA1	24476	3606	1.78E-11	11	-0.031	0.005	Maternal effect for dead at 21 days (litter)
LA1	24476	3606	1.96E-10	10	0.054	0.008	Drip loss
LA1	24476	3606	4.34E-10	9	-1.107	0.177	Meat quality
LA1	24476	3606	8.23E-10	9	1.271	0.207	Maternal ability
LA1	24476	3606	1.18E-09	9	1.035	0.170	Carcass quality
LA1	24476	3606	2.49E-09	9	-1.480	0.248	Total number born
