# Supplementary material for: Loss of function mutations in essential genes cause embryonic lethality in pigs
Source: PLoS Genet. 2019 Mar 15;15(3):e1008055. doi: 10.1371/journal.pgen.1008055 (PMC6436757; doi:10.1371/journal.pgen.1008055)
Supplement: S16 Table — (PDF) [file pgen.1008055.s035.pdf]

Table S16: Association analysis for LA2, LA3, and DU1 carriers. AA represents the number of non-carriers in the database, while AB represents the number of carrier animals. Results show no or very little evidence for heterozygote advantage for the LA2, LA3, and DU1 lethal haplotypes.

Source	AA	AB	P value	-log10 (Pvalue)	effect	se	Trait
LA2	25726	2356	0.000187	4	-0.042	0.011	Number of teats
LA2	25726	2356	0.001559	3	0.098	0.031	Meat percentage
LA2	25726	2356	0.002196	3	-0.041	0.013	Total number born
LA3	26763	1319	5.00E-09	8	-1.640	0.28	Carcass quality
LA3	26763	1319	8.01E-05	4	0.009	0.002	Intramuscular fat
LA3	26762	1319	0.000164	4	-0.053	0.014	Drip loss
LA3	26766	1319	0.000164	4	0.249	0.066	Meat quality
DU1	9699	1077	0.000153	4	0.05289	0.01396	Front Leg Pasterns
DU1	9699	1077	0.00032	3	-0.1291	0.03585	Belly meat percentage
DU1	9699	1077	0.000795	3	-0.1412	0.04208	Loin Depth
DU1	9699	1077	0.001631	3	0.01282	0.004068	Weaning weight 21 Days
