# Supplementary material for: Loss of function mutations in essential genes cause embryonic lethality in pigs
Source: PLoS Genet. 2019 Mar 15;15(3):e1008055. doi: 10.1371/journal.pgen.1008055 (PMC6436757; doi:10.1371/journal.pgen.1008055)
Supplement: S17 Table — (PDF) [file pgen.1008055.s036.pdf]

Table S17: Litter information for purebred and crossbred litters in the Landrace population. Table provides the number of records, the average, and standard deviation (stdev) within each of the two mating classes.

	#Number of records	#Average	#Stdev
**Landrace purebred matings**	23,132	14.176	3.499
**Landrace crossbred litters***	58,027	14.381	3.595

* The landrace sow is mated with a Large White boar.
