# Supplementary material for: Loss of function mutations in essential genes cause embryonic lethality in pigs
Source: PLoS Genet. 2019 Mar 15;15(3):e1008055. doi: 10.1371/journal.pgen.1008055 (PMC6436757; doi:10.1371/journal.pgen.1008055)
Supplement: S19 Table — (PDF) [file pgen.1008055.s038.pdf]

Table S19: WGS samples including breed and coverage information.

Sample	Population	Coverage
D405	Duroc	16.40
L528	Landrace	13.37
D149	Landrace	11.95
D576	Duroc	13.51
D715	Duroc	12.73
L844	Landrace	17.06
L965	Landrace	16.39
D257	Duroc	13.14
L807	Landrace	10.83
D837	Duroc	8.59
D927	Duroc	12.76
L766	Landrace	11.34
D126	Duroc	16.04
L174	Landrace	10.67
L483	Landrace	13.37
D902	Duroc	16.82
D458	Duroc	17.47
D024	Duroc	7.21
D834	Duroc	15.96
L120	Landrace	17.04
L362	Landrace	15.99
D023	Duroc	17.04
D255	Duroc	13.33
D338	Duroc	15.94
D477	Duroc	16.57
D478	Duroc	14.49
D833	Duroc	6.65
D929	Duroc	12.65
L051	Landrace	11.05
L394	Landrace	10.91
L572	Landrace	12.14
L169	Landrace	10.75
L330	Landrace	10.39
L990	Landrace	9.67
D569	Duroc	14.72
L078	Landrace	13.76
D427	Duroc	15.28
L827	Landrace	15.60
D352	Duroc	15.26
L779	Landrace	15.13
L787	Landrace	15.26
D797	Duroc	14.76
L879	Landrace	16.67
D000	Duroc	14.93
L196	Landrace	15.26
L105	Landrace	15.60
D431	Duroc	8.77
D432	Duroc	10.26
D433	Duroc	8.80
D434	Duroc	9.78
D435	Duroc	8.07
D436	Duroc	8.64
D437	Duroc	11.23
D438	Duroc	10.52
D439	Duroc	9.41
