# Supplementary material for: Loss of function mutations in essential genes cause embryonic lethality in pigs
Source: PLoS Genet. 2019 Mar 15;15(3):e1008055. doi: 10.1371/journal.pgen.1008055 (PMC6436757; doi:10.1371/journal.pgen.1008055)
Supplement: S20 Table — (PDF) [file pgen.1008055.s039.pdf]

Table S20: Heterozygous variants identified in the *POLR1B* and *TADA2A* RNA-seq expression data. The presence of heterozygous variants supports the lack of genomic imprinting, and expression of both the maternal and paternal allele.

Chromosome	Position	Ref/Alt	Gene	VEP Annotation	Heterozygous sample	Reference observed	Alternate observed
3	43949498	G/T	*POLR1B*	3’ UTR	711604	2	1
3	43952858	C/T	*POLR1B*	Missense	761156	9	4
12	38903760	T/C	*TADA2A*	Synonymous	787897	3	2
